# Optimization of supraclavicular lymph node clinical target volume delineation in high-risk breast cancer: a single center experience and recommendation

**DOI:** 10.1186/s12885-023-11596-6

**Published:** 2023-11-29

**Authors:** Li Li, Hongyan Zhang, Linwei Wang, Conghua Xie, Haijun Yu, Yahua Zhong

**Affiliations:** 1 Department of Radiation and Medical Oncology, Zhongnan Hospital of Wuhan University, #169, Donghu Road, Wuchang District, Wuhan, 430071 Hubei China; 2 Hubei Cancer Clinical Study Center, #169, Donghu Road, Wuchang District, Wuhan, Hubei 430071 China; 3 Hubei Key Laboratory of Tumor Biological Behaviors, #169, Donghu Road, Wuchang District, Wuhan, Hubei 430071 China

**Keywords:** Breast cancer, Supraclavicular lymph nodes, Radiotherapy, Clinical target volume delineation

## Abstract

**Background:**

Prophylactic irradiation of supraclavicular lymph node drainage areas can improve the regional control rate of lymph node-positive or lymph node-negative disease but a locally-advanced stage breast cancer, and it can reduce breast cancer-related mortality. However, many controversies exist in the clinical target volume delineation of supraclavicular lymph node drainage in patients with breast cancer.

**Methods:**

We retrospectively analyzed 42 patients with breast cancer and supraclavicular lymph node metastasis at our hospital between January 2017 and December 2021. Among these cases, 32 were locally advanced and 10 were stage IV at initial treatment. A patient with breast cancer who did not undergo dissection of the supraclavicular and infraclavicular lymph nodes at our hospital was selected as a standard patient. A contrast-enhanced computed tomography (CT) scan for positioning was used as a template image, and blood vessels, muscles, and bony landmarks were used as references for positioning. The metastatic supraclavicular lymph nodes were identified in all enrolled patients and projected into the template CT images.

**Results:**

The metastastic pattern of supraclavicular lymph node in breast cancer was proposed: distribution along the posterolateral border of the internal jugular vein (medial supraclavicular group) and along the transverse jugular vein (lateral supraclavicular group). We theorized that the lateral and posterior borders of the clinical target volume in the supraclavicular region should include the lymph nodes in the posterior triangle of the neck (level V) in high-risk individuals. If the metastatic axillary lymph node is extensive, then the superior border of the supraclavicular region should be moved upward appropriately.

**Conclusions:**

This study analyzed patients with breast cancer and supraclavicular lymph node metastasis at initial treatment, explored the metastastic pattern of supraclavicular lymph node, and applied anatomical knowledge to further optimize the target volume delineation of supraclavicular lymph node drainage area in high-risk breast cancer.

**Supplementary Information:**

The online version contains supplementary material available at 10.1186/s12885-023-11596-6.

## Introduction

Breast cancer is the most common malignant tumour in female individuals. Its morbidity and mortality are increasing annually [1]. Randomized clinical trials show that regional lymph node radiotherapy can improve the local control rate of breast cancer and reduce breast cancer-related mortality in patients with one to three positive lymph nodes [2]. In patients with breast cancer and risk factors, such as multiple axillary lymph node metastasis, lymphovascular invasion, and a high histological grade, the recurrence rate of supraclavicular lymph nodes is > 20%. Prophylactic irradiation of regional lymph nodes can reduce this to < 10% [2–4].

The second most common sites of local recurrence in breast cancer are supraclavicular lymph nodes, second only to the chest wall [5]. Therefore, radiation therapy to the supraclavicular region is crucial for the local control of breast cancer. Currently, many controversies remain in the clinical target volume (CTV) delineation of supraclavicular lymph nodes [5–8]. Many studies are based on the recurrence pattern of supraclavicular lymph nodes after local treatment; however, lymphatic drainage in the supraclavicular region is disrupted after radiotherapy or surgery. Therefore, it does not accurately reflect the pattern of lymph node metastasis in the supraclavicular region. This study aimed to retrospectively analyze patients with breast cancer and supraclavicular lymph node metastasis at initial treatment, to explore the metastastic pattern of supraclavicular lymph node, and to apply anatomical knowledge to further optimize the CTV delineation of supraclavicular region in breast cancer.

## Methods

### Patient cohort

The inclusion criteria were as follows: 1) age ≥ 18 years, 2) patients with breast cancer and supraclavicular lymph node metastasis at initial treatment, and 3) diagnosed by computed tomography (CT), magnetic resonance imaging (MRI), positron emission imaging (PET), or pathological examination. The exclusion criteria were as follows: 1) recurrent supraclavicular lymph node metastasis, 2) lack of imaging data, and 3) prior supraclavicular radiation or systemic therapy. 42 breast cancer patients who meeting the inclusion and exclusion criteria at our hospital between January 2017 and December 2021 were retrospectively analyzed. Among these cases, 32 were locally advanced stage and 10 were stage IV at initial treatment. All imaging data were reassessed by two experienced radiologists. The following diagnostic criteria for supraclavicular lymph node metastasis were used [6, 9]: 1) lymph nodes with a short diameter of > 1 cm; 2) ring enhancement, central necrosis, or extracapsular invasion; and 3) hypermetabolism on PET-CT or supraclavicular lymph node metastasis confirmed by pathological biopsy. This study has been approved by the Ethics Committee of Zhongnan Hospital of Wuhan University. The ethical code is 2,020,020. Due to the retrospective nature of the study, the informed consent requirement was waived by the Ethics Committee of Zhongnan Hospital of Wuhan University.

### Mapping

A patient with breast cancer who did not undergo dissection of the supraclavicular and infraclavicular lymph nodes at our hospital was selected as a standard patient. The Body Mass Index (BMI) of the patient is 22.8, she underwent breast-conserving surgery combined with sentinel lymph node biopsy, and the pathological staging was pT1cN0M0. The orientation of the CT scan for positioning was the same as that in adjuvant radiotherapy after breast cancer mastectomy: supine with the head neutral, the upper arm of the affected side abducted above the head and the upper limb of the healthy side positioned at the side of the body. The slice thickness of CT scan is 3 mm. The contrast-enhanced CT scan was used as a template image, and blood vessels, muscles, and bony landmarks were used as references for positioning. The principal investigator identified the metastatic supraclavicular lymph nodes in all enrolled patients and projected these into the template CT images. All lymph nodes were circled with a diameter of 4 mm. The anatomical site where the centre of the lymph node was located was marked for lymph nodes > 5 mm.

## Results

### Patient characteristics

**Fig. 1:**
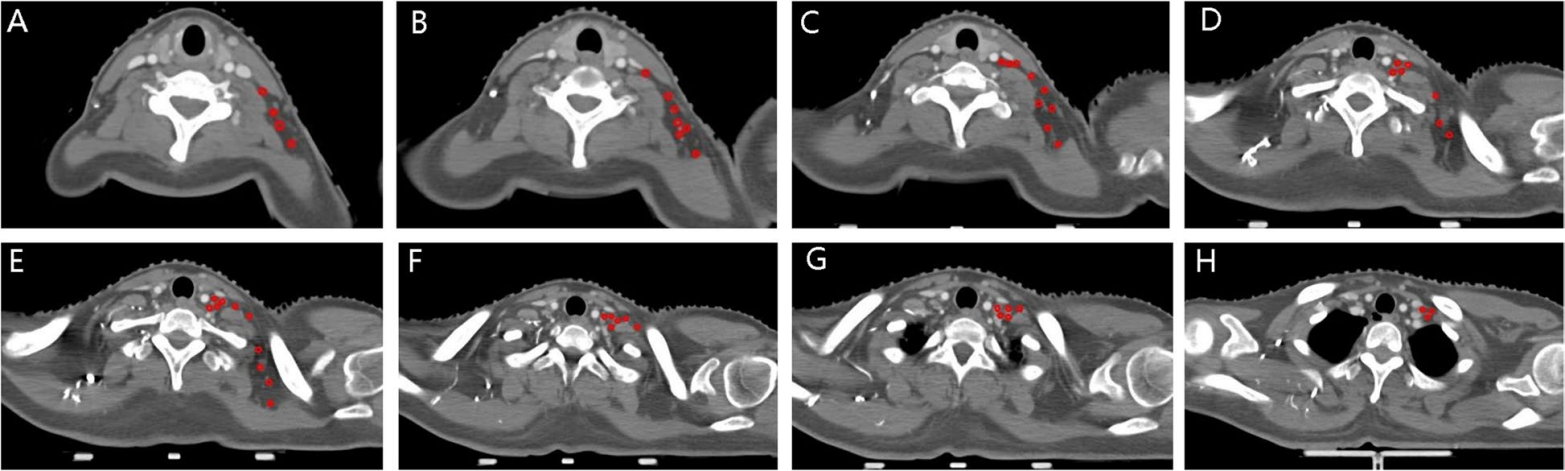
**A**-**H** The centre of positive supraclavicular lymph nodes were identified and projected into the template CT images with red circles of 4 mm diameter

The baseline characteristics of the 42 patients were as following (Table 1). A total of 109 lymph nodes were identified and mapped in 42 patients with breast cancer and supraclavicular lymph node metastasis (Fig. 1A-H) who met the inclusion and exclusion criteria.

**Table 1:** The baseline characteristics of the 42 patients

Characteristic	Value
Age (y)	
Median	48
Range	25–72
Stage of initial diagnosis	
M0	10 (23.8%)
M1	32 (76.2%)
Histological grade (WHO)	
I	0
II	23 (54.8%)
III	19 (45.2%)
Axillary lymph node involved	
Level I	35(83.3%)
Level II	32(76.2%)
Level III	12(28.6%)

### Distribution of supraclavicular lymph nodes based on vascular markers

#### Distribution along the posterolateral border of the internal jugular vein (medial supraclavicular group)

**Fig. 2:**
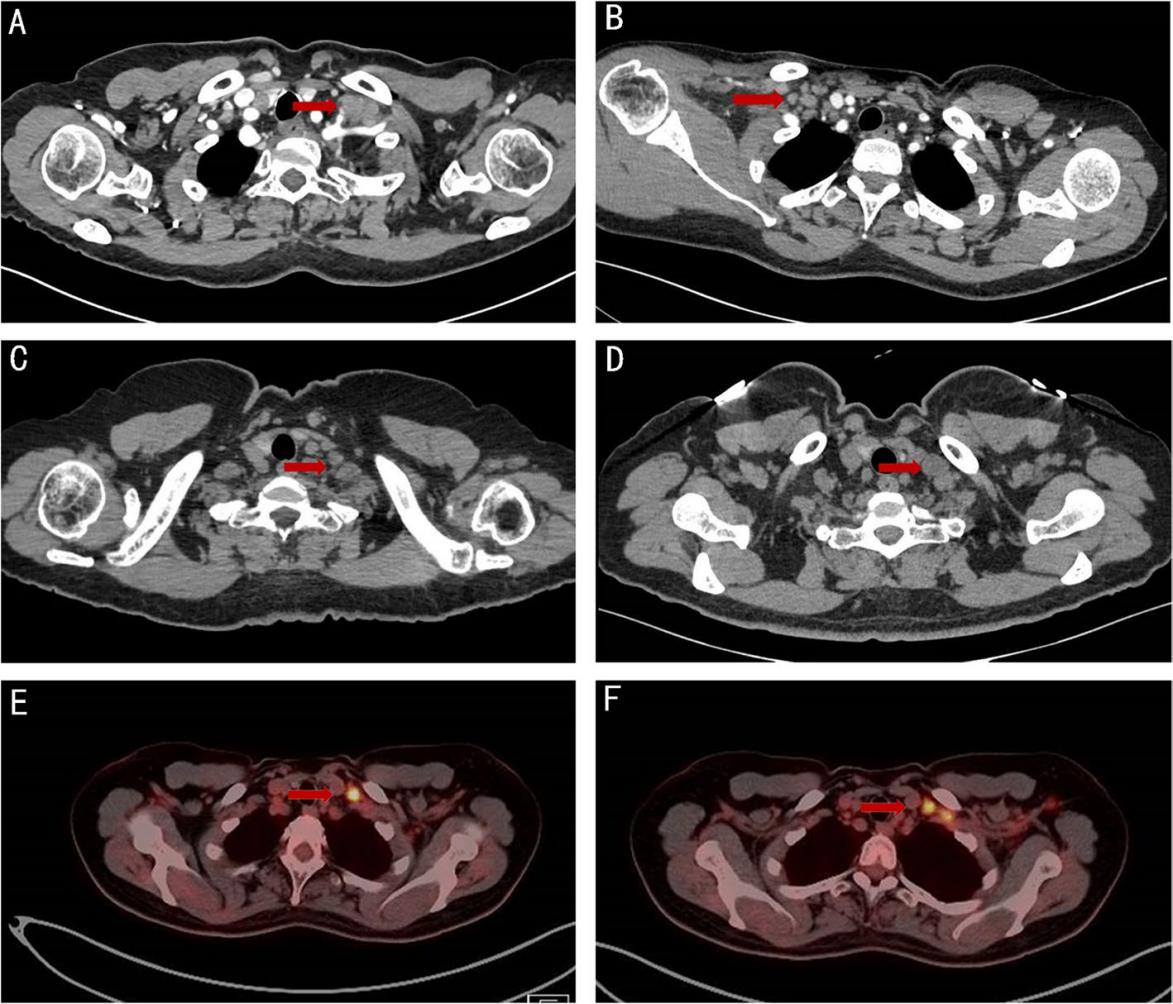
**A**-**F** The positive lymph nodes distributing along the posterolateral border of the internal jugular vein (medial supraclavicular group)

**Fig. 3:**
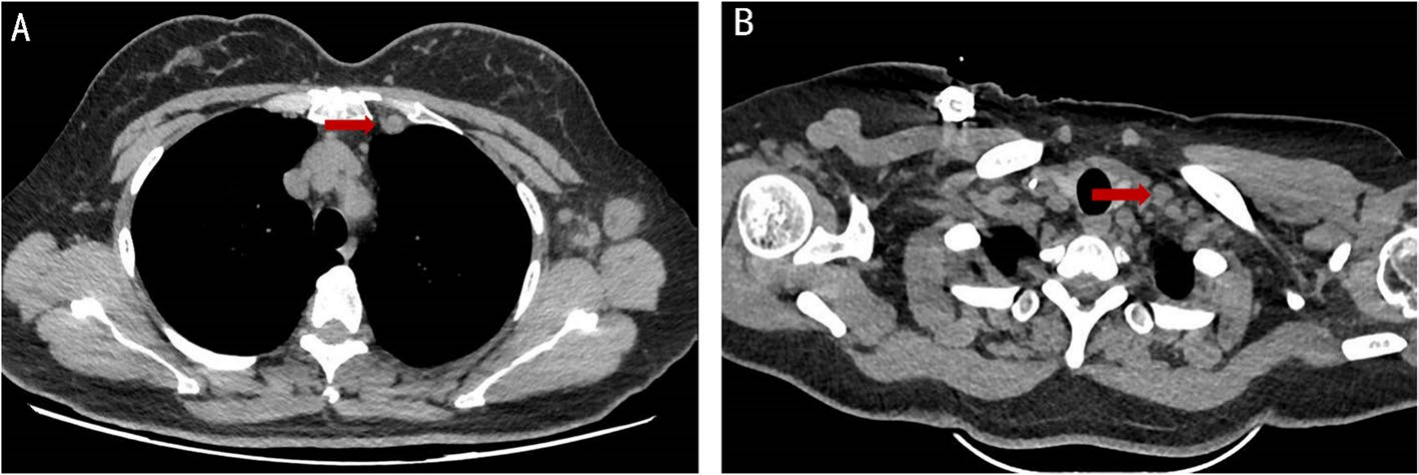
**A**-**B** This figure shows one of the important pathway for supraclavicular lymph node metastasis: it can spread upward to the supraclavicular region through the internal mammary lymphatic chain (**A** Metastatic supraclavicular lymph node in the jugular vein angle area, **B** Highly suspicious lymph node in the internal mammary region at the same time)

All metastatic lymph nodes were distributed along the posterolateral border of the internal jugular vein, mainly in the venous angle region. No metastatic lymph nodes were present in the anteromedial portion of the internal jugular vein and carotid artery (Fig. 2A-F); in a small number of patients, metastatic lymph nodes appeared in the jugular vein angle area, highly suspicious lymph nodes can be observed in the internal mammary region at the same time (Fig. 3A-B).

#### Distribution along the transverse jugular vein (lateral supraclavicular group)

**Fig. 4:**
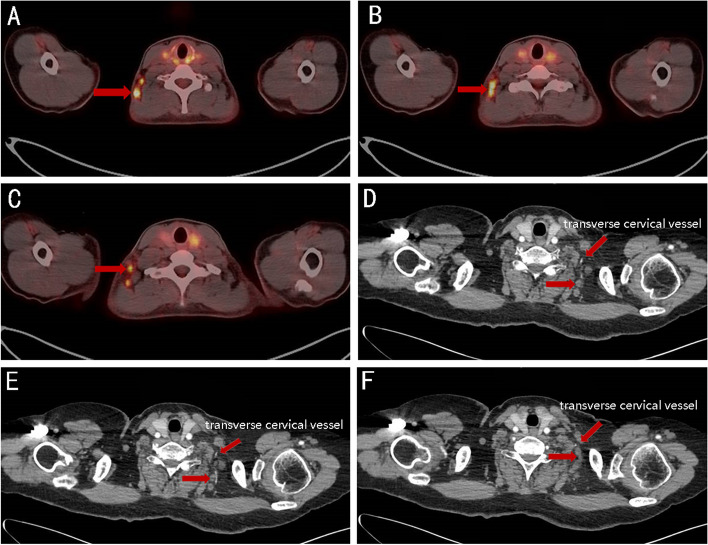
**A**-**F** Metastatic lymph nodes in the posterior triangle of the neck (level V, lateral supraclavicular group) in cases of extensive axillary lymph node metastasis

**Fig. 5:**
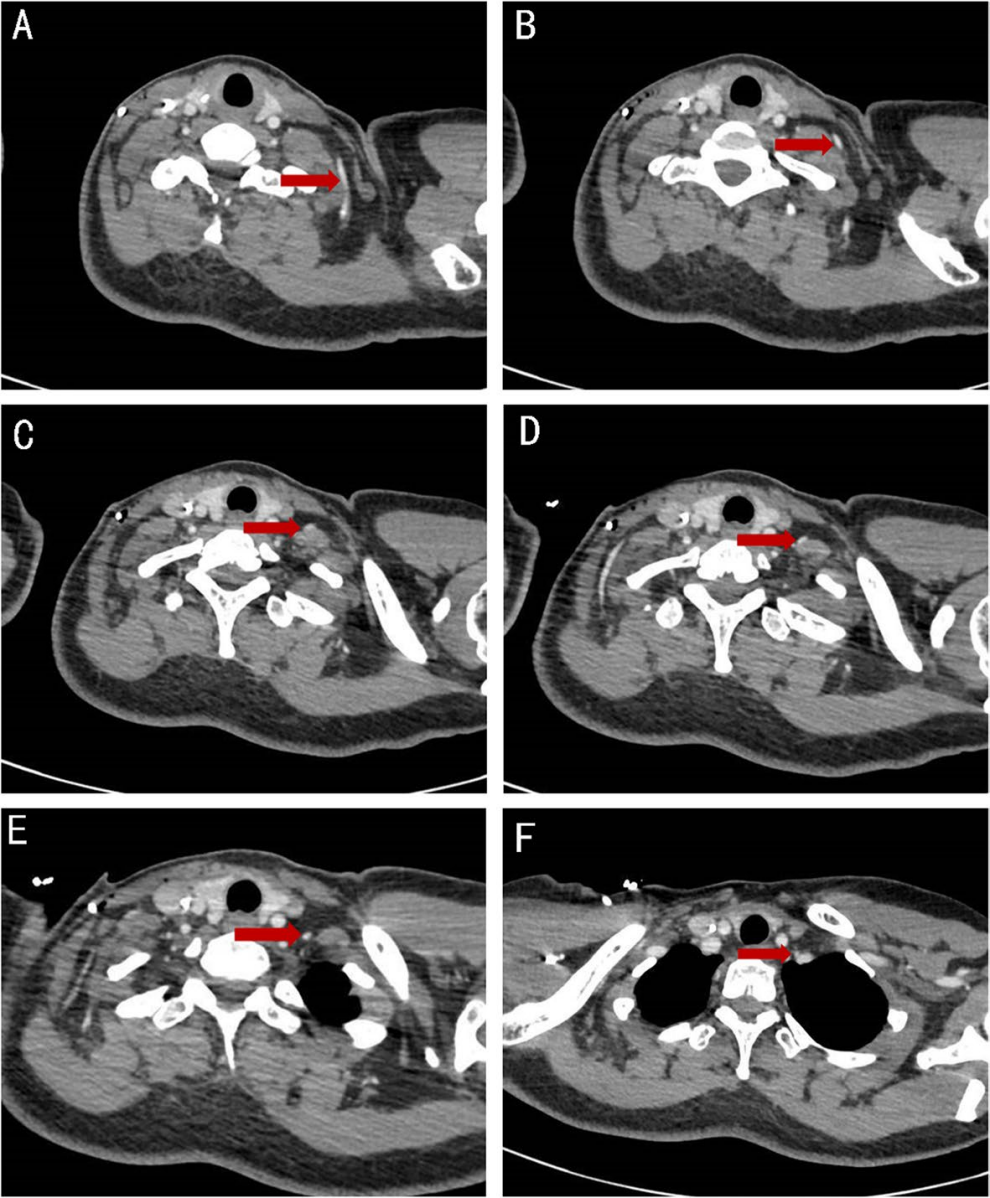
**A**-**F** The anatomical course of transverse jugular vessels

Metastatic lymph nodes were often present in the posterior triangle of the neck (level V, lateral supraclavicular group) in cases of extensive axillary lymph node metastasis (Fig. 4A-F). Most metastatic lymph nodes in this area were distributed along the transverse jugular vessels (Fig. 5A-F). The outer border did not exceed the omohyoideus muscle, and the inner border did not exceed the scalene muscle bundle.

### Target volume delineation of supraclavicular lymph nodes based on lymph node distribution

**Fig. 6:**
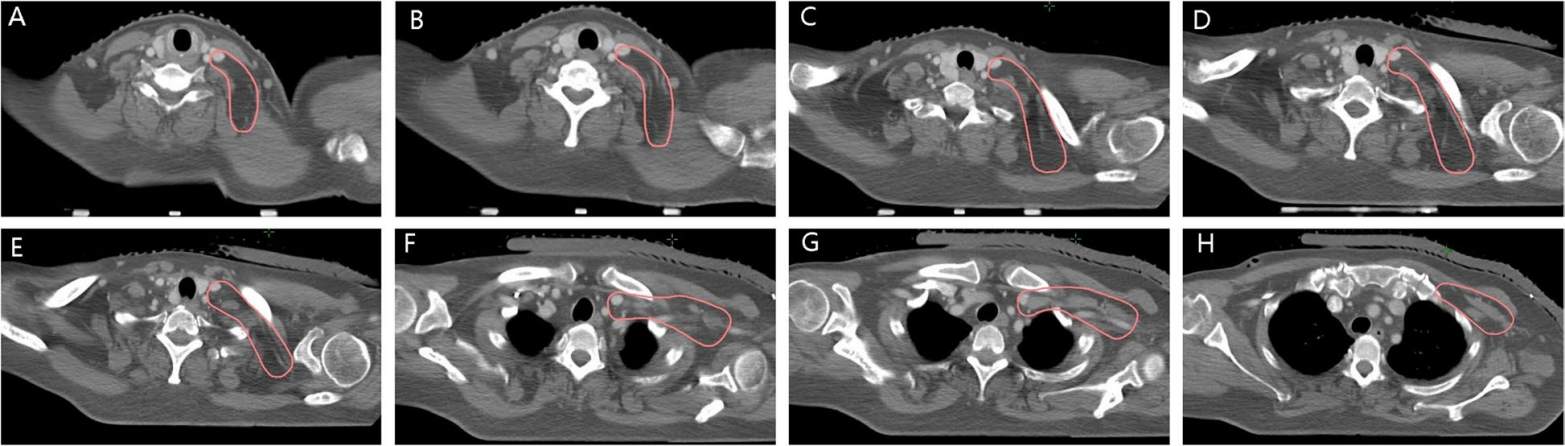
**A**–**H** Clinical target volume delineation of supraclavicular region based on metastatic lymph nodes distribution

The superior border comprised the lower edge of the cricoid cartilage. If the metastatic axillary lymph node is extensive, then the superior border of the supraclavicular region should be moved upward appropriately. The inferior border comprised the level where the clavicular head disappeared. The medial border comprised the scalene muscle bundle and lateral border of cervical vessels (carotid artery and internal jugular vein). The lateral border comprised the medial border of the sternocleidomastoid/omohyoideus muscle and superficial layer of the deep cervical fascia. The anterior border was posterior to the internal jugular vein, and the posterior border was anterior to the trapezius muscle (Fig. 6A–H).

## Discussion

Many clinical studies on breast cancer confirm the significance of the prophylactic irradiation of supraclavicular lymph node drainage areas. It not only reduces the local recurrence rate but also significantly improves overall survival [2–4]. With the development of radiotherapy technology, radiotherapy of the supraclavicular region has changed from traditional two-dimensional radiotherapy to three-dimensional conformal radiotherapy, followed by the modern intensity-modulated radiotherapy. Radiotherapy planning with CT simulation for positioning is increasingly applied in radiotherapy for breast cancer. Currently, universal guidelines for delineating supraclavicular lymph node target volumes include those of the Radiation Therapy Oncology Group (RTOG) [10], a relatively accessible reference template for delineating breast/chest wall and regional lymph node radiotherapy targets in clinical trials. Relevant retrospective clinical studies include those of Mayo Clinic and the Chinese Academy of Medical Sciences Cancer Hospital [6, 7]. This study aimed to be distinct in terms of the enrolled population, distribution of lymph nodes, and CTV delineation of supraclavicular lymph nodes in breast cancer.

We enrolled patients with breast cancer who were newly diagnosed with supraclavicular lymph node metastasis. All patients had not received local or systemic treatment at the time of enrollment. Thus, the distribution of lymph nodes truly reflected the biological behaviour of supraclavicular lymph node metastasis in breast cancer. Other studies [5–7, 11], including those of Mayo Clinic, the Netherlands, and the Chinese Academy of Medical Sciences Cancer Hospital, included not only patients with breast cancer and supraclavicular lymph node metastases but also some patients with supraclavicular lymph node recurrence after initial treatment, particularly axillary/supraclavicular lymph node dissection. The metastatic pattern of supraclavicular lymph nodes differ between untreated and newly treated patients, because the latter experience a disrupted lymphatic return. Therefore, we optimized the enrolled population to accurately reflect the rule of supraclavicular lymph node metastasis in breast cancer.

The lymph node metastasis rule is based on blood vessel distribution. Previous studies have characterized the general region of supraclavicular lymph node metastasis in breast cancer but have not explained the metastasis rule in depth. Jumping metastasis of lymph nodes is rare in breast cancer. The most common route of metastasis is from the axillary to the subclavicular and then supraclavicular region. Extensive axillary lymph node metastasis significantly increases the probability of lymph node metastasis in the supraclavicular region. We observed that metastatic lymph nodes were present at the back of the neck (level V) in cases of extensive axillary lymph node metastasis. Notably, this exceeded RTOG’s definition for supraclavicular metastasis [10]. The subclavian vein drains the blood flow of the transverse jugular vein. The distribution of many axillary lymph nodes along the subclavian vein will cause a portion of the lymph fluid to flow backward to the back of the neck (level V), enlarging the lymph nodes in this area. This is the anatomical basis of lateral supraclavicular lymph node metastasis. Additionally, enlarged lymph nodes were occasionally present in the venous angle region (medial supraclavicular group) when clinical data suggested significantly enlarged lymph nodes in the internal mammary region. The internal mammary vein drains to the subclavian vein, and the lymph nodes in the venous angle can be transferred along the internal mammary lymph chain. This is an anatomical basis of medial supraclavicular lymph node metastasis. Based on the data at our institution, observed clinical phenomena, and anatomical knowledge, this study proposed two courses of supraclavicular lymph node metastasis to reinterpret the rule of supraclavicular lymph node metastasis in breast cancer: distribution along the posterolateral border of the internal jugular vein (medial supraclavicular group) and distribution along the transverse cervical vessels (lateral supraclavicular group).

Based on the above mentioned rules of supraclavicular lymph node metastasis, we optimized the delineation of supraclavicular lymph node target volume in breast cancer. The superior border comprised the lower edge of the cricoid cartilage. If the metastatic axillary lymph node is extensive, then the superior border of the supraclavicular region should be moved upward appropriately. The inferior border comprised the level where the clavicular head disappeared, followed by the target volume of chest wall/breast. The medial border comprised the scalene muscle bundle and lateral border of cervical vessels (carotid artery and internal jugular vein). The lateral border comprised the medial border of the sternocleidomastoid/omohyoideus muscle and superficial layer of the deep cervical fascia. The anterior border was posterior to internal jugular vein, and the posterior border was anterior to the trapezius muscle border. According to randomized controlled trials by the European Organization for Research and Treatment of Cancer 22922 [12] and National Cancer Institute of Canada Clinical Trials Group MA.20 [13], addition of regional lymph node irradiation on the basis of whole breast irradiation reduces regional lymph node recurrence. This emphasizes the importance of adequate coverage of nodal regions. The main difference between our recommended target volume and that of the RTOG [10] is our inclusion of level V (behind the neck) to the lateral and posterior borders, which was consistent with the recommendation of the Chinese Academy of Medical Sciences Cancer Hospital [7]. If the axillary lymph node metastasis is extensive, the superior border of the supraclavicular area should be moved upward appropriately.

This study had the following limitations: first, we employed a retrospective design. Thus, selection bias may have occurred. Second, the sample size was small. Thus, it may not fully reflect the rule of lymph node metastasis in the supraclavicular region. Third, the enrolled patients were either locally advanced or metastatic breast cancer, therefore, the conclusion of the suggested improved delineation is applicable to high-risk patients, not to low or moderate risk population. Lastly, some cases of clinically significant regional lymph node metastases may not have been detected or may have been detected only after the occurrence of distant metastases. Thus, future large-scale, prospective clinical trials are needed to further guide treatment.

### Supplementary Information

**Additional file 1: Supplementary Figure.** Metastatic lymph nodes in supraclavicular region (the red arrow represent positive lymph node).

## Data Availability

The datasets used and/or analysed during the current study available from the corresponding author on reasonable request.

## Abbreviations

CTV: Clinical target volume
CT: Computed tomography
PET: Positron emission tomography
RTOG: Radiation Therapy Oncology Group
