# Supplementary material for: Optimization of supraclavicular lymph node clinical target volume delineation in high-risk breast cancer: a single center experience and recommendation
Source: BMC Cancer. 2023 Nov 29;23:1168. doi: 10.1186/s12885-023-11596-6 (PMC10688076; doi:10.1186/s12885-023-11596-6)
Supplement: Supplementary file 1 — Additional file 1: Supplementary Figure. Metastatic lymph nodes in supraclavicular region (the red arrow represent positive lymph node). [file 12885_2023_11596_MOESM1_ESM.pdf]

Supplementary Figure: Metastatic lymph nodes in supraclavicular region (the red arrow represent positive lymph node)

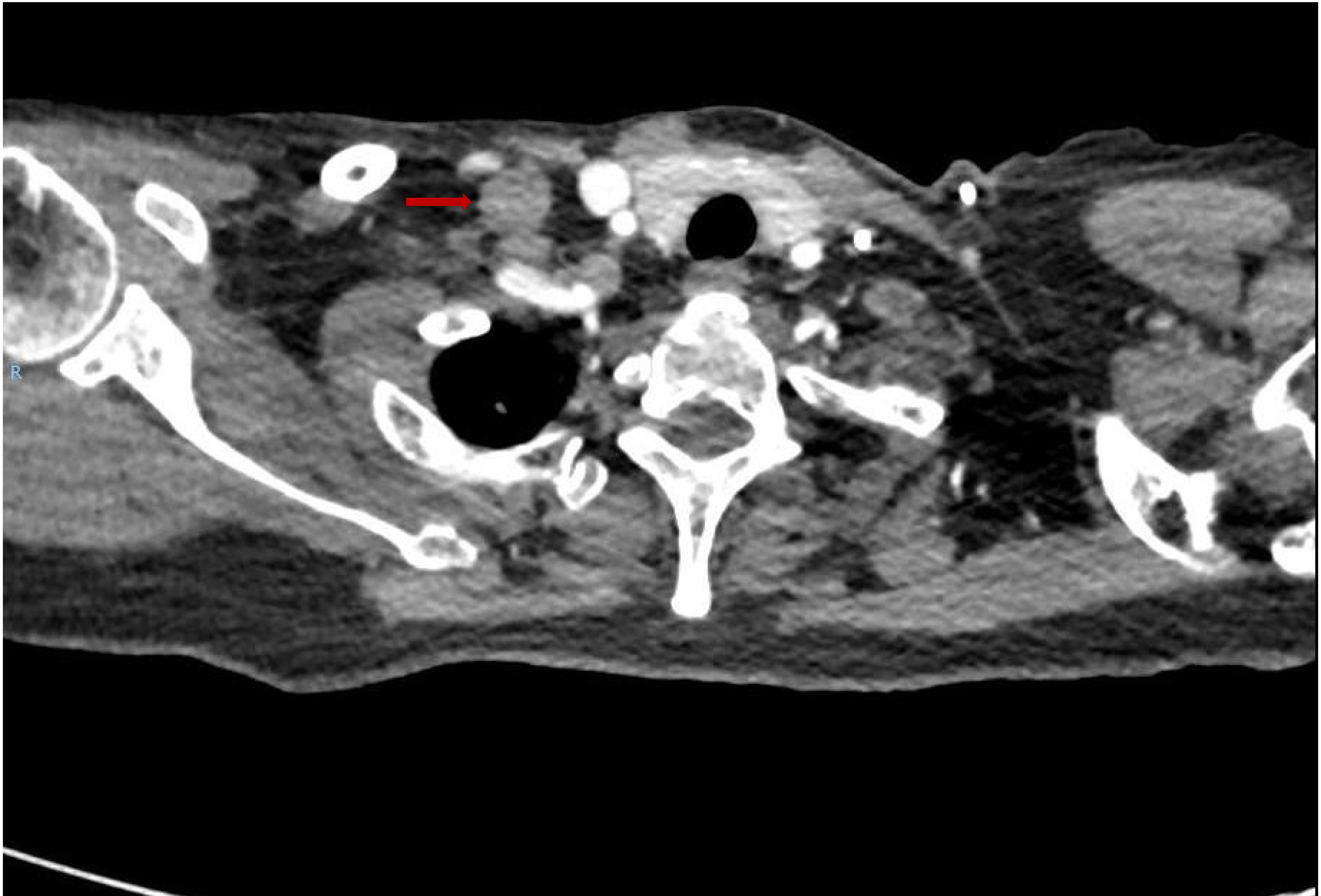

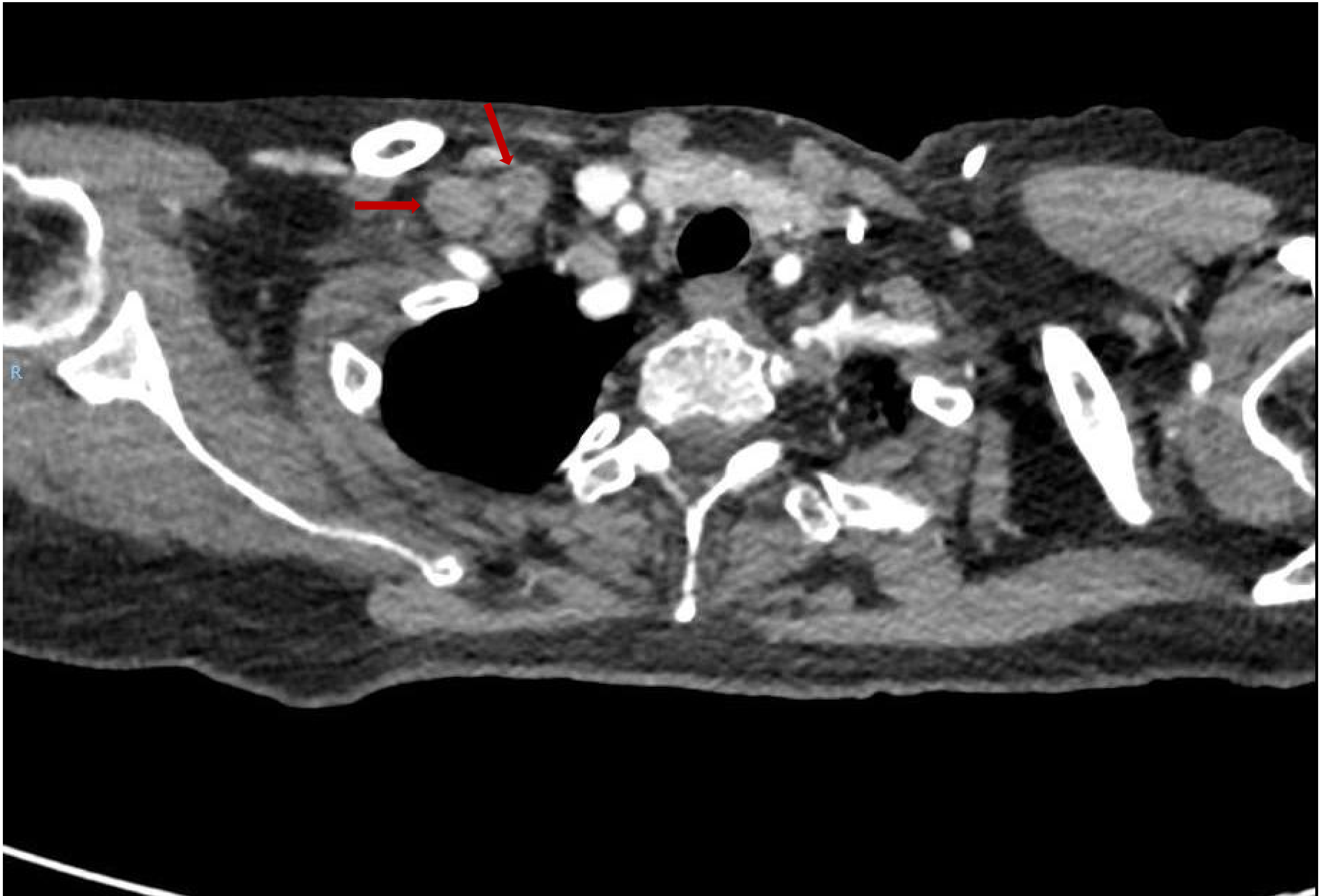
R

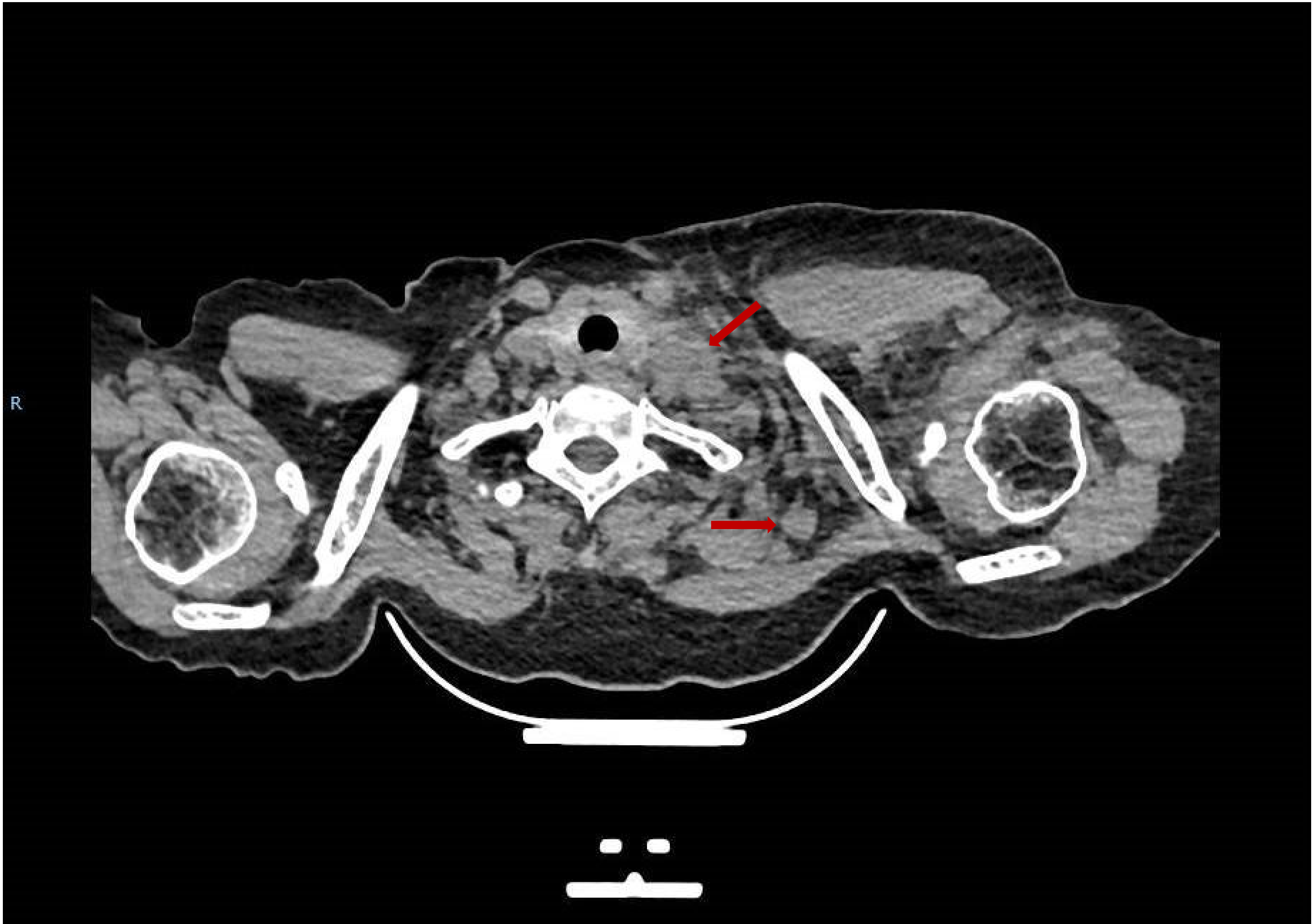
R

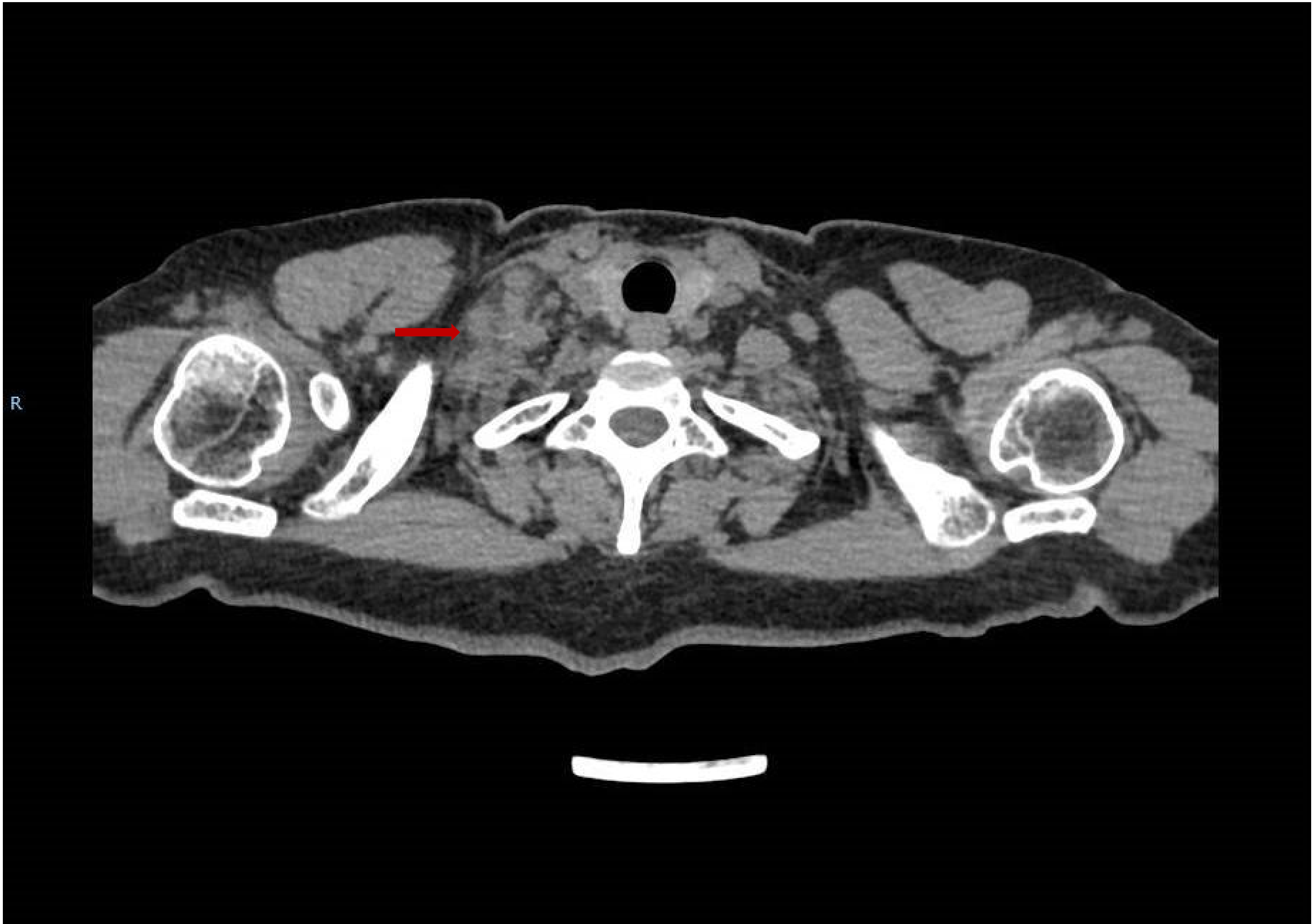
R

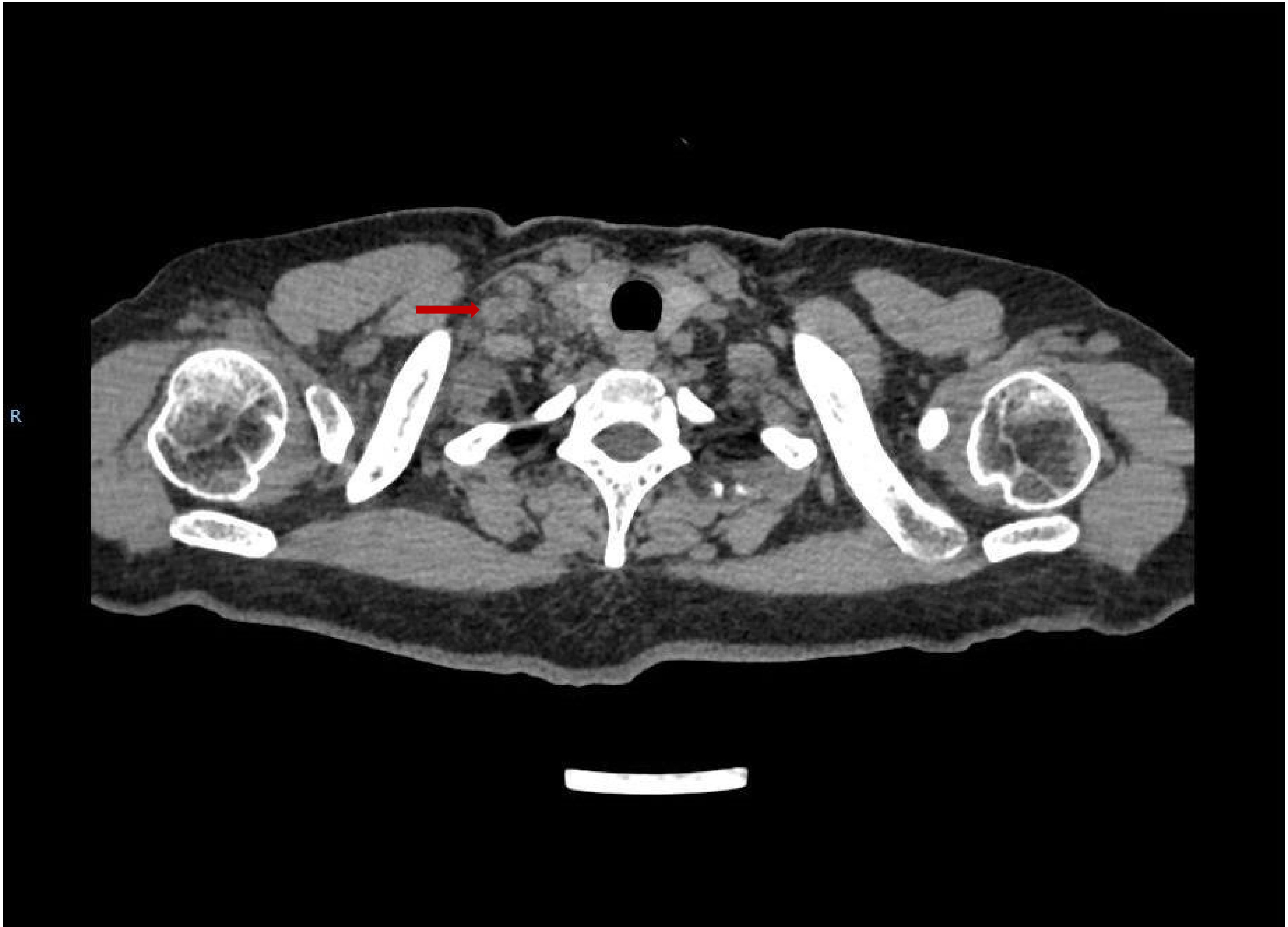
R

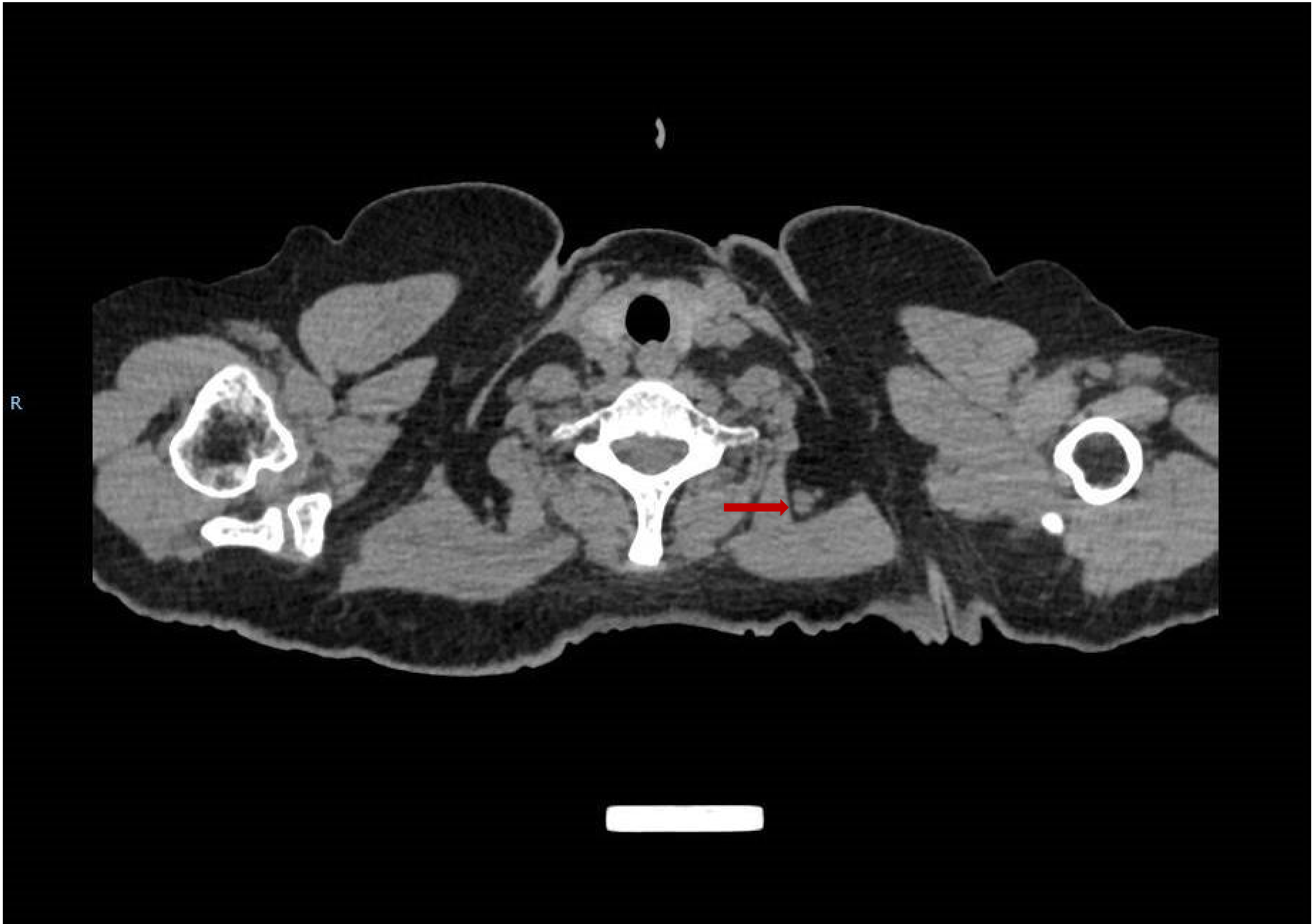
R

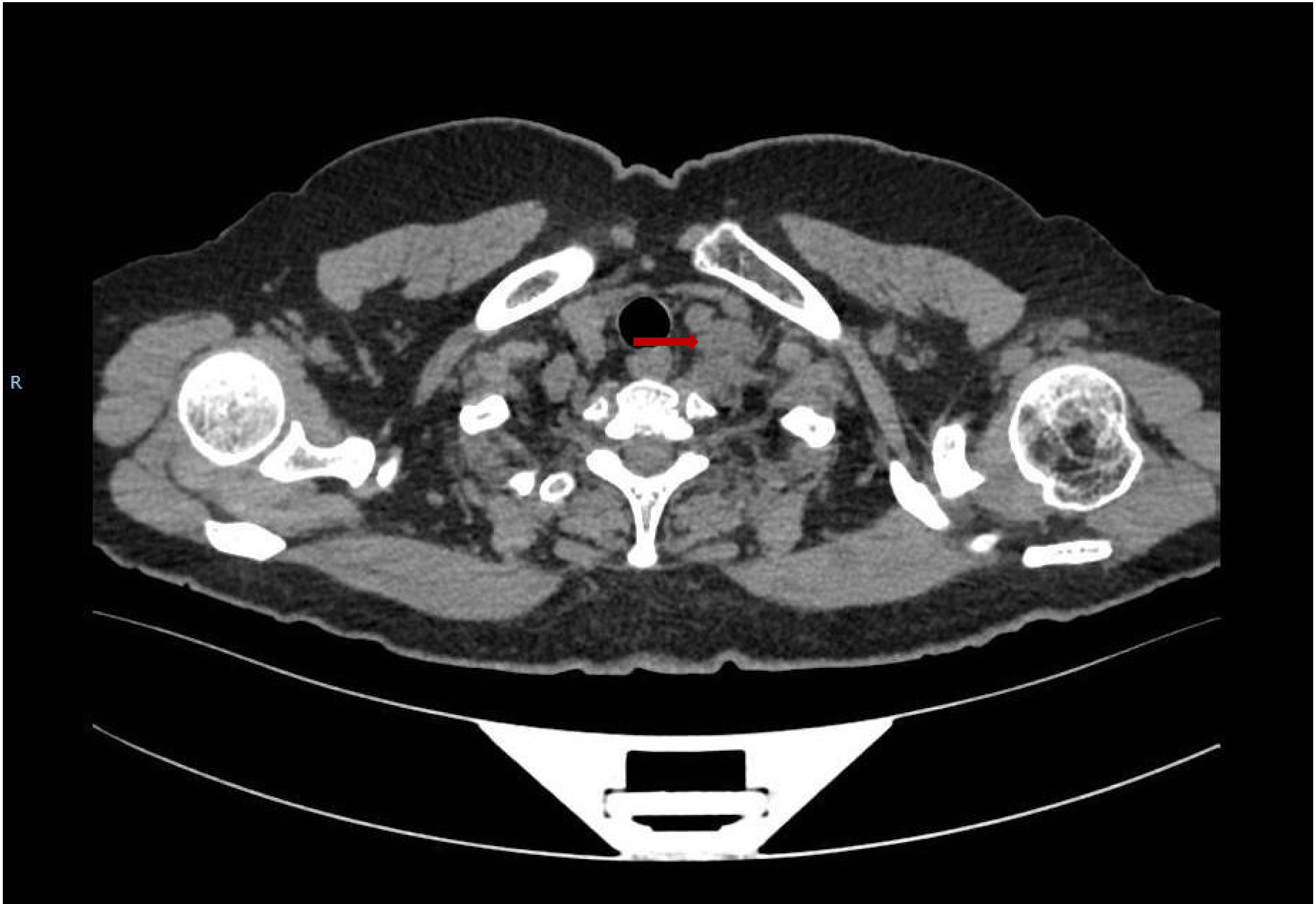
R

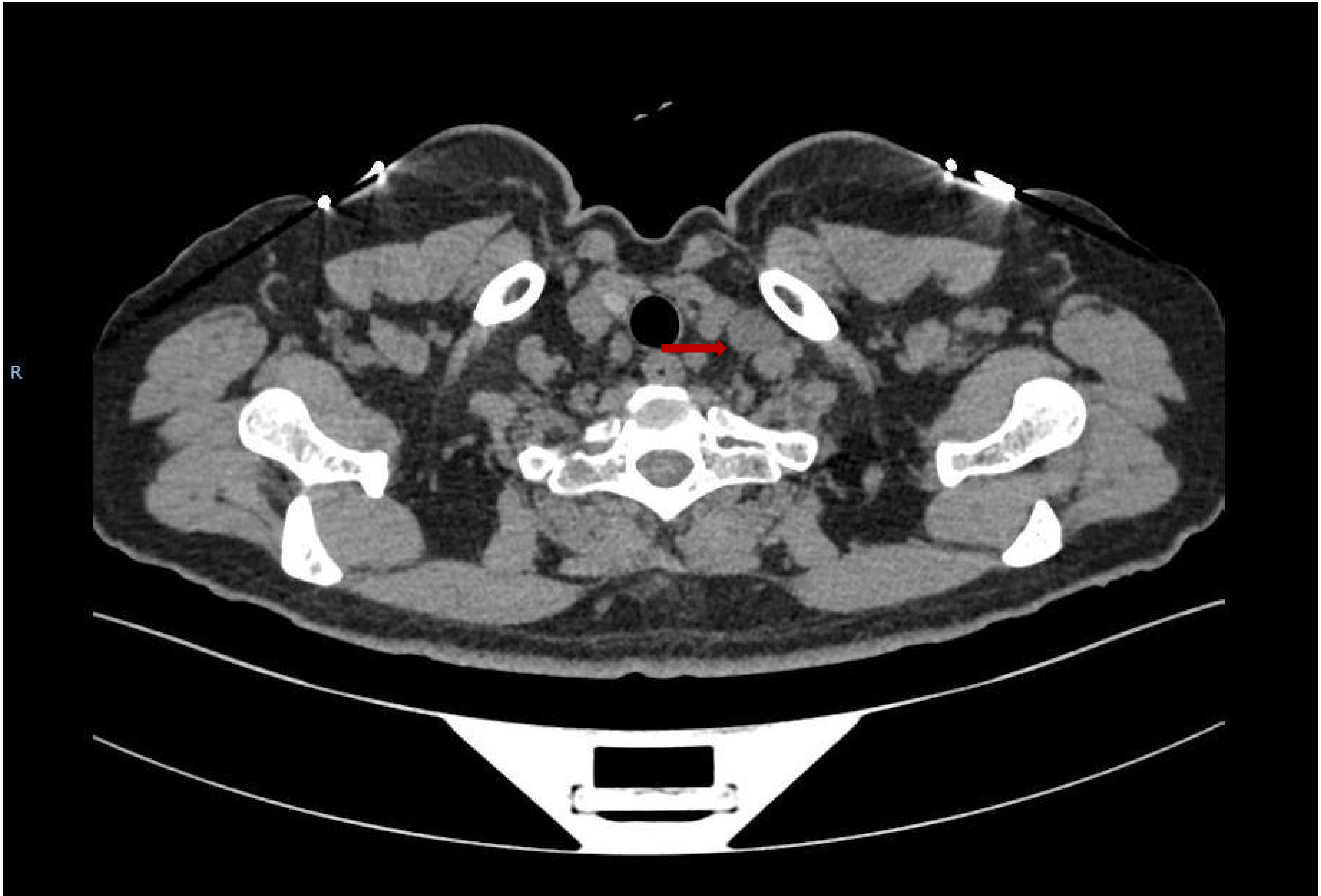
R

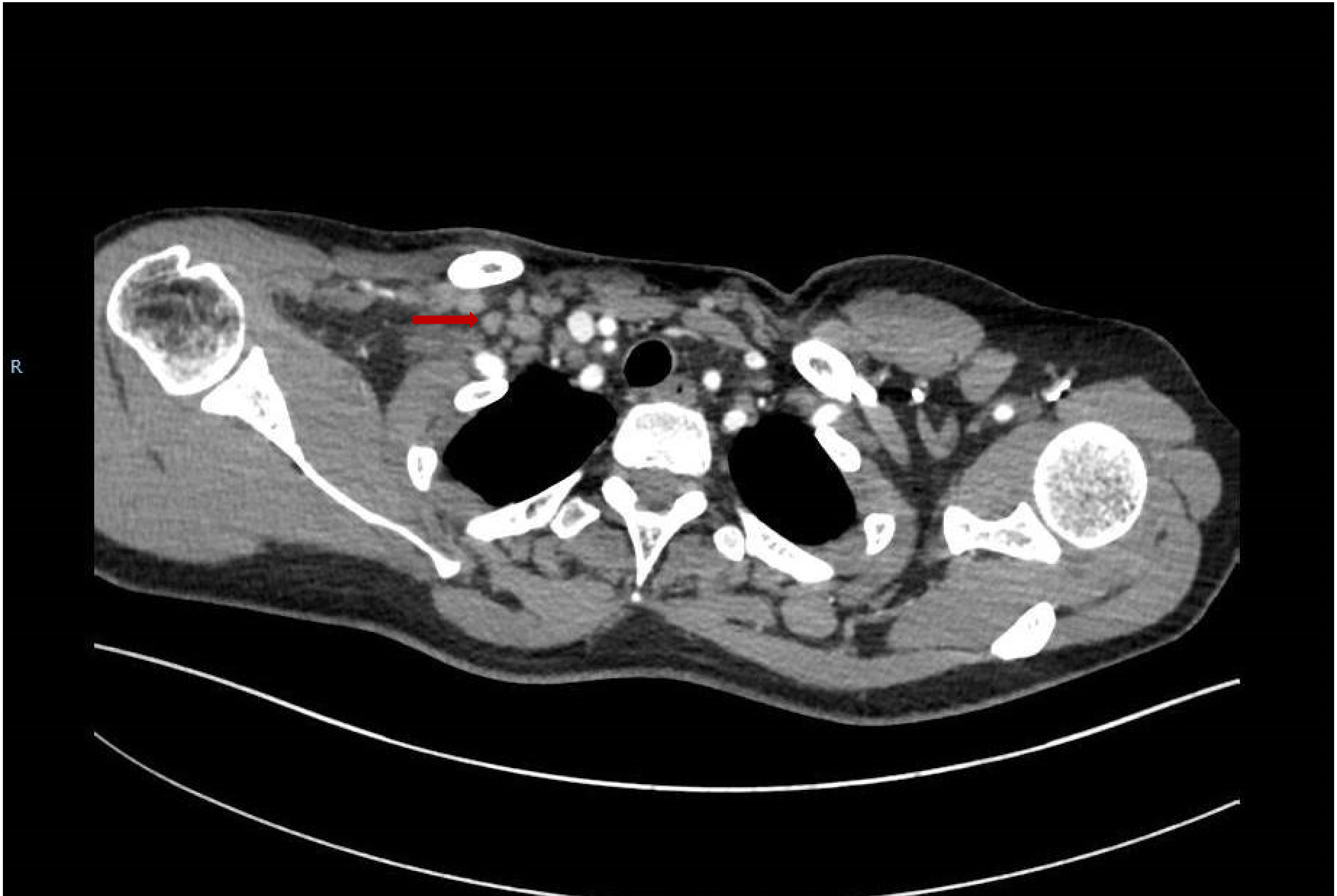
R

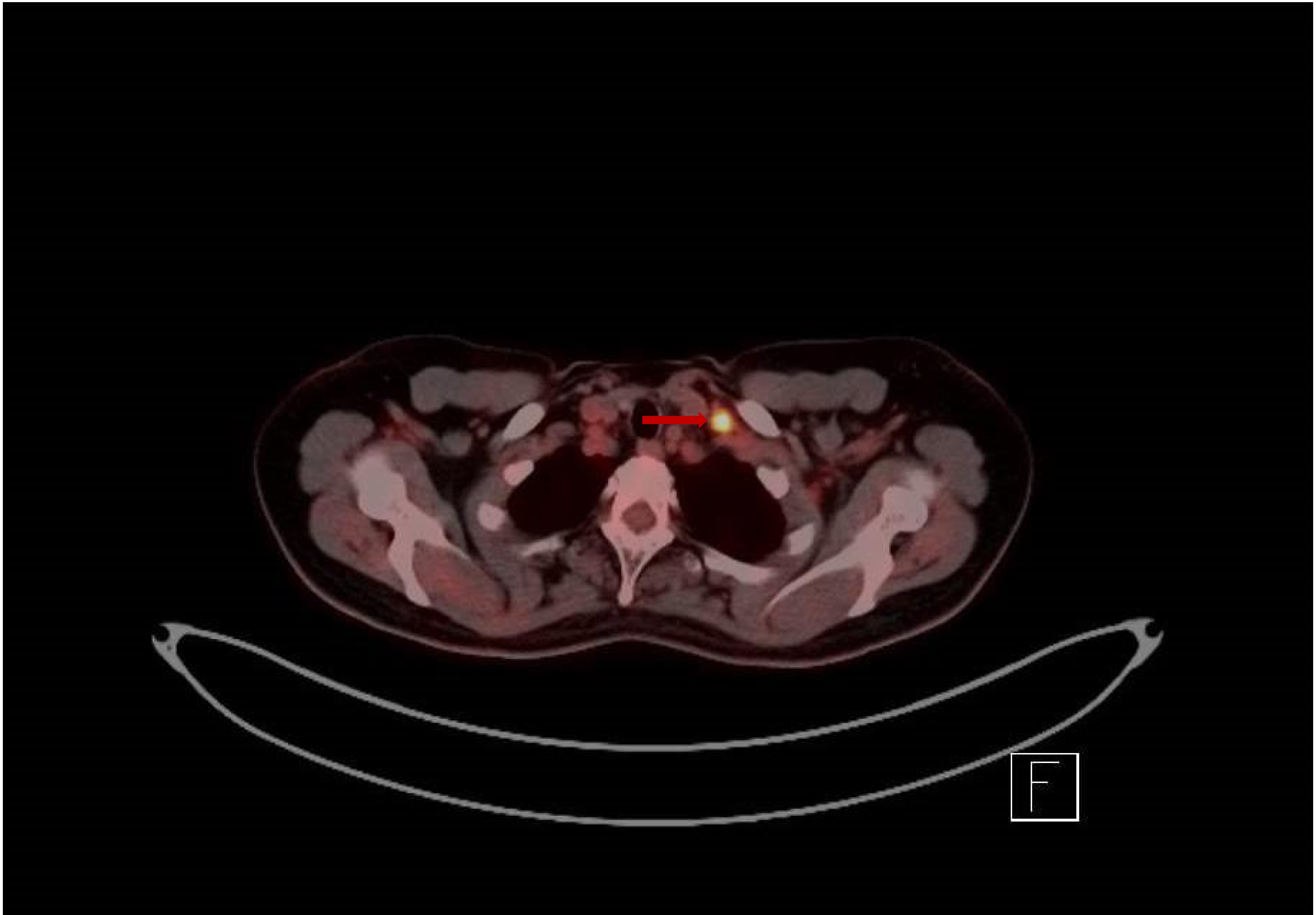
F

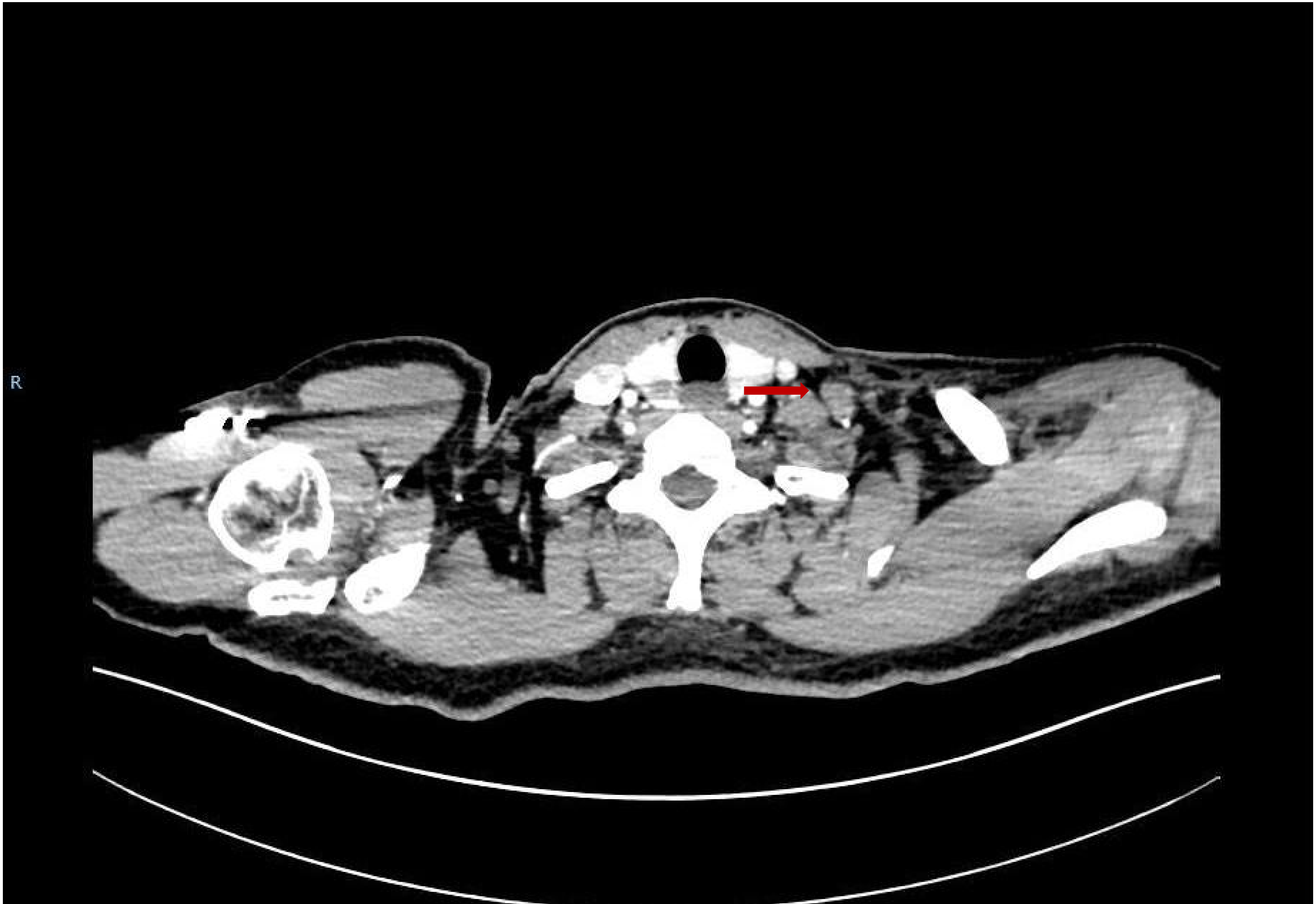
R

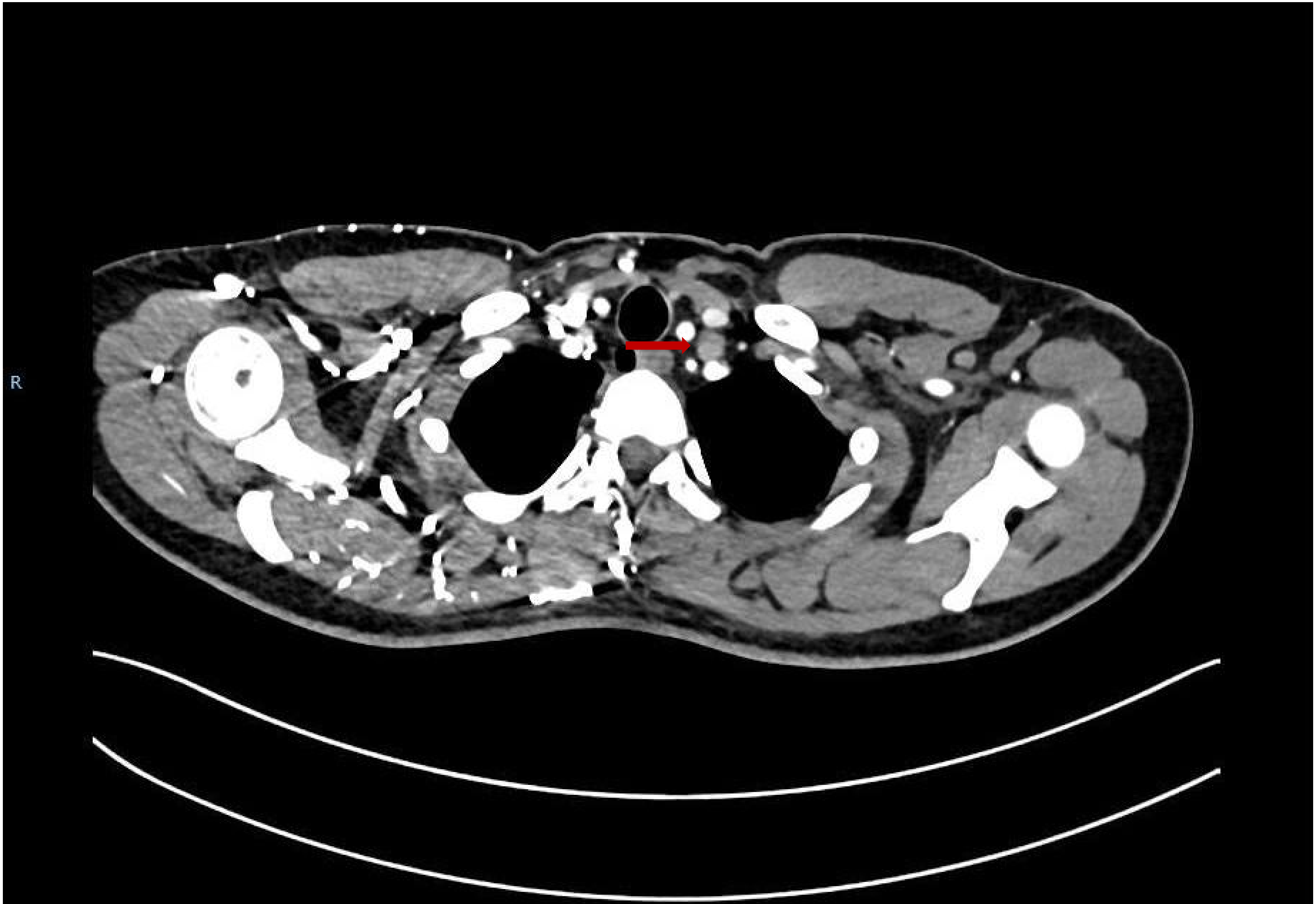
R

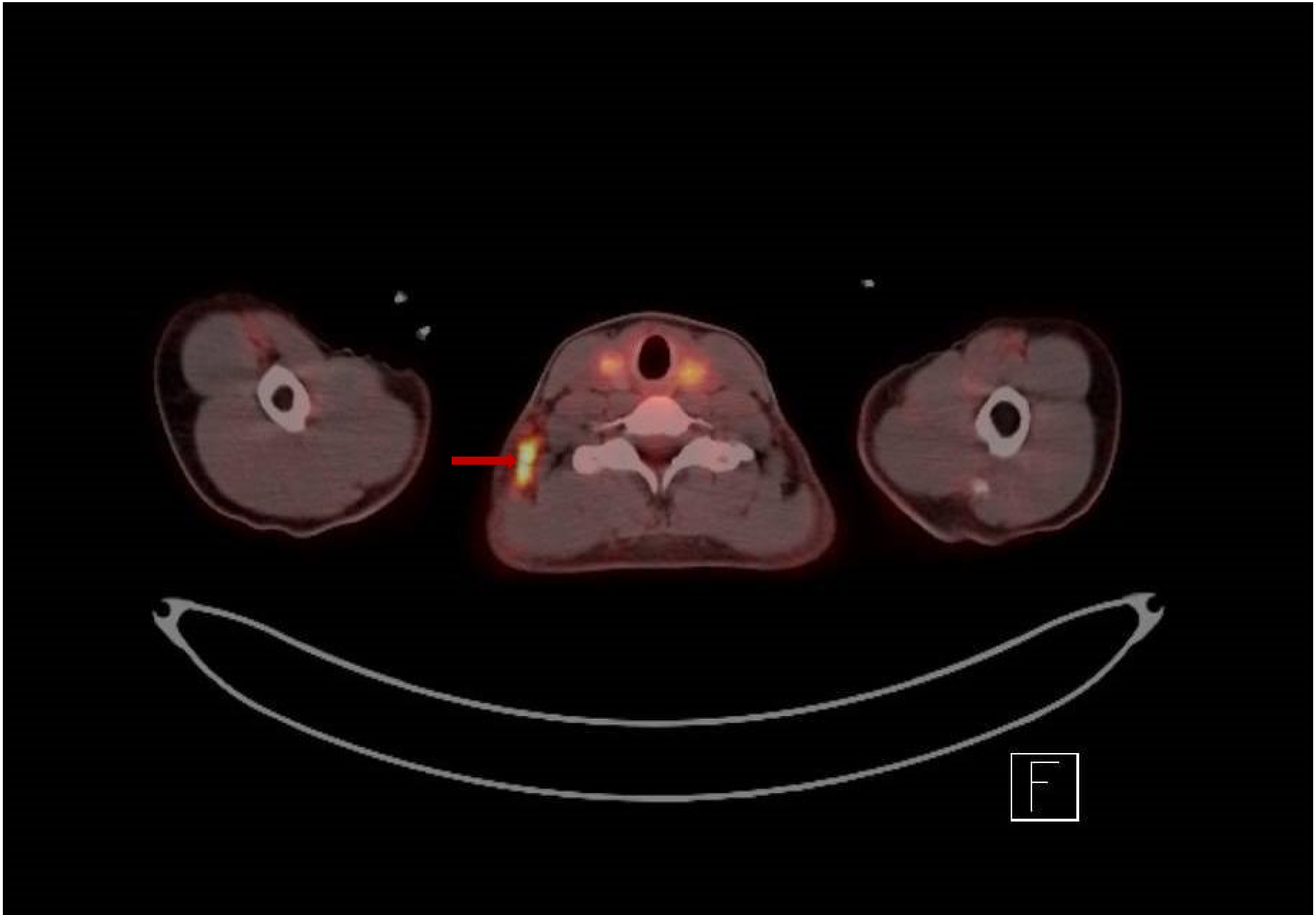
F

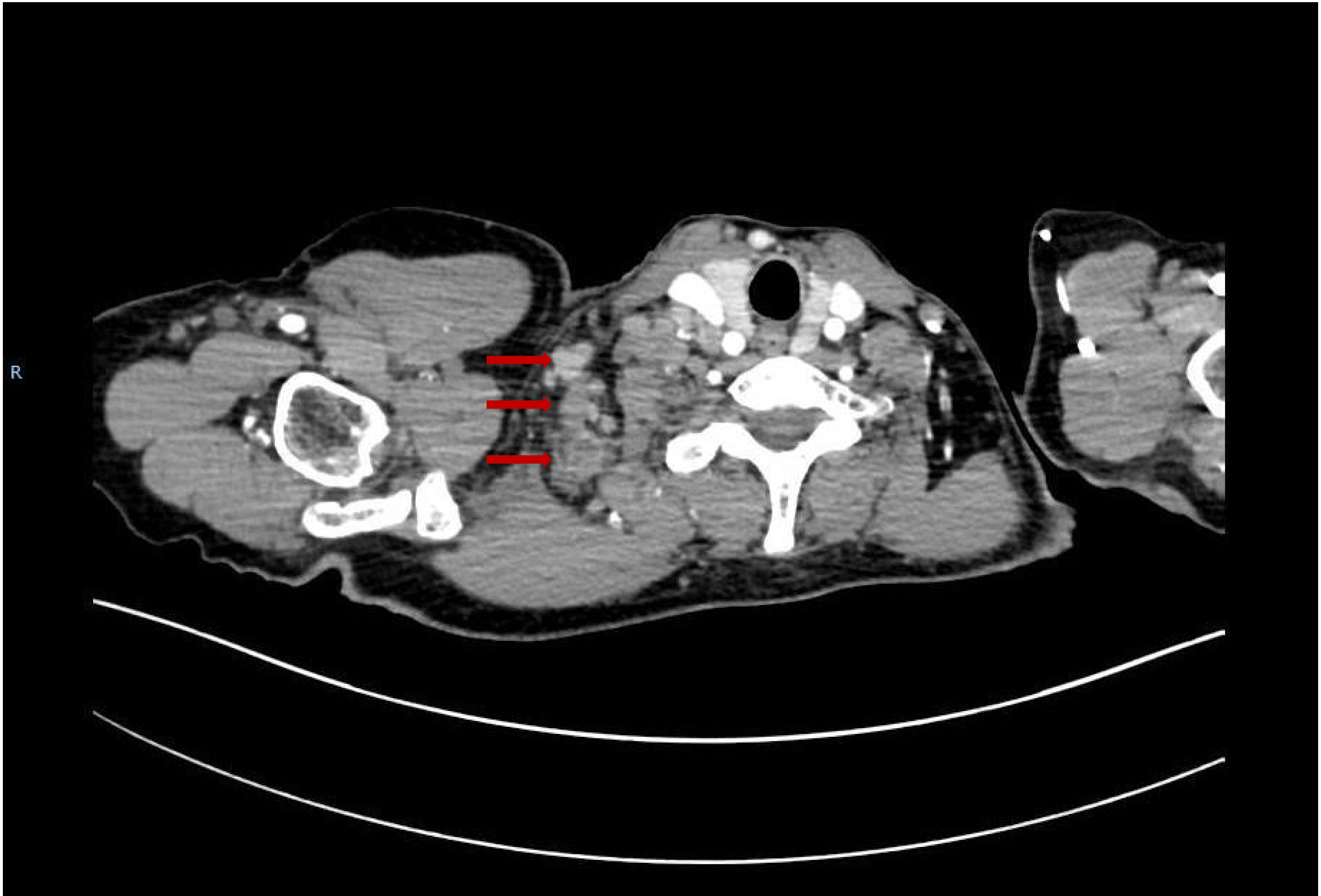
R

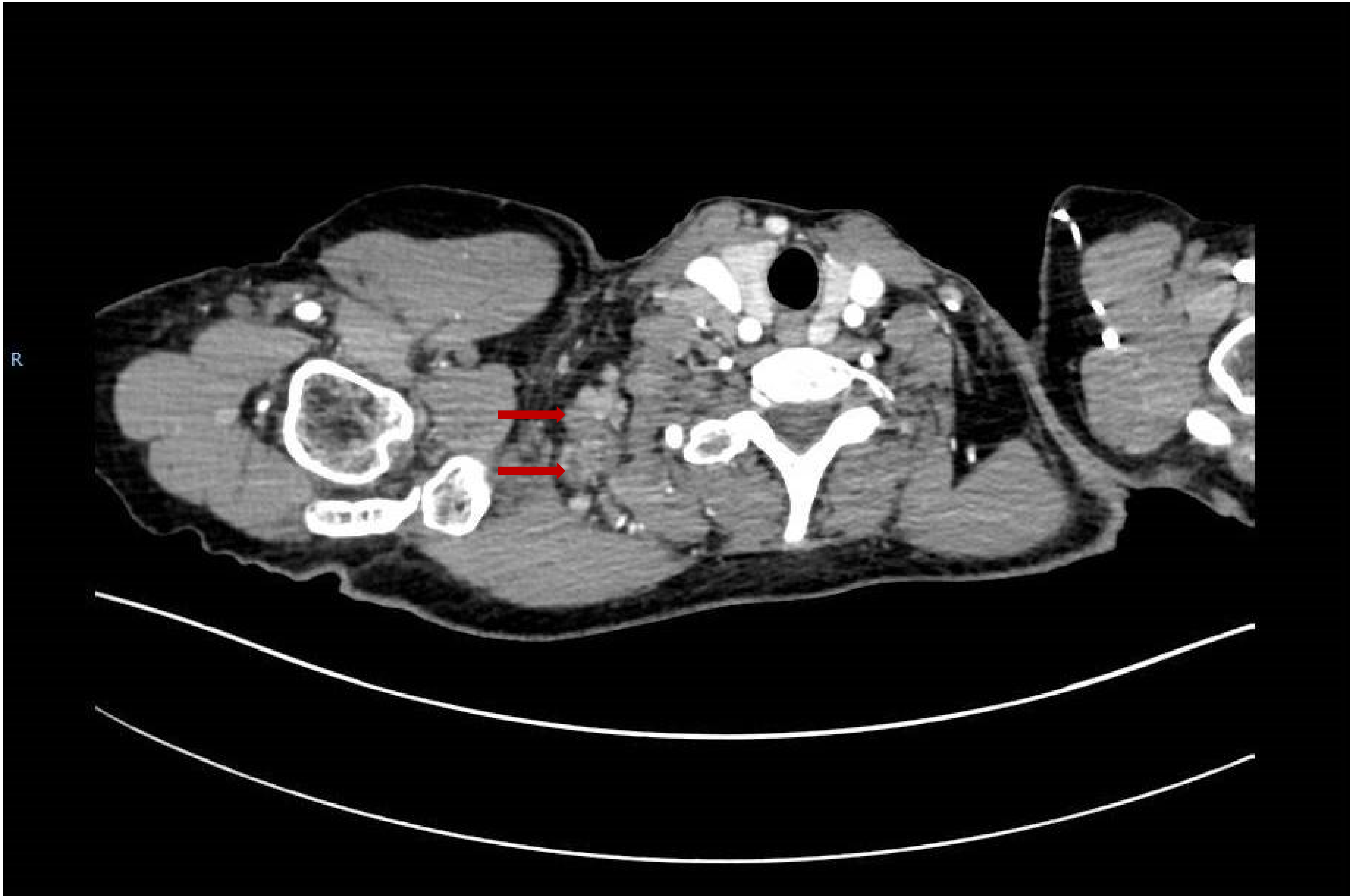
R

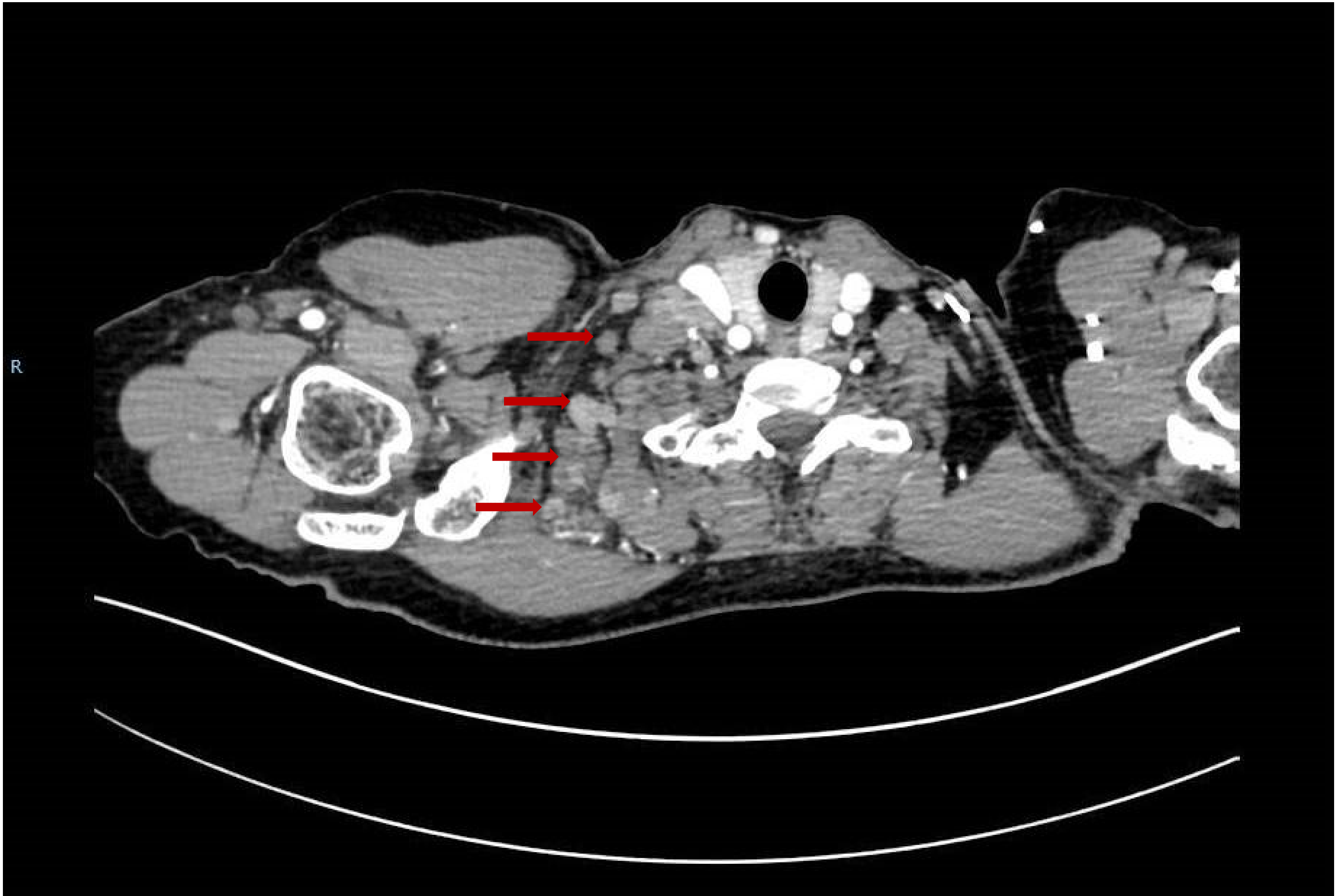
R

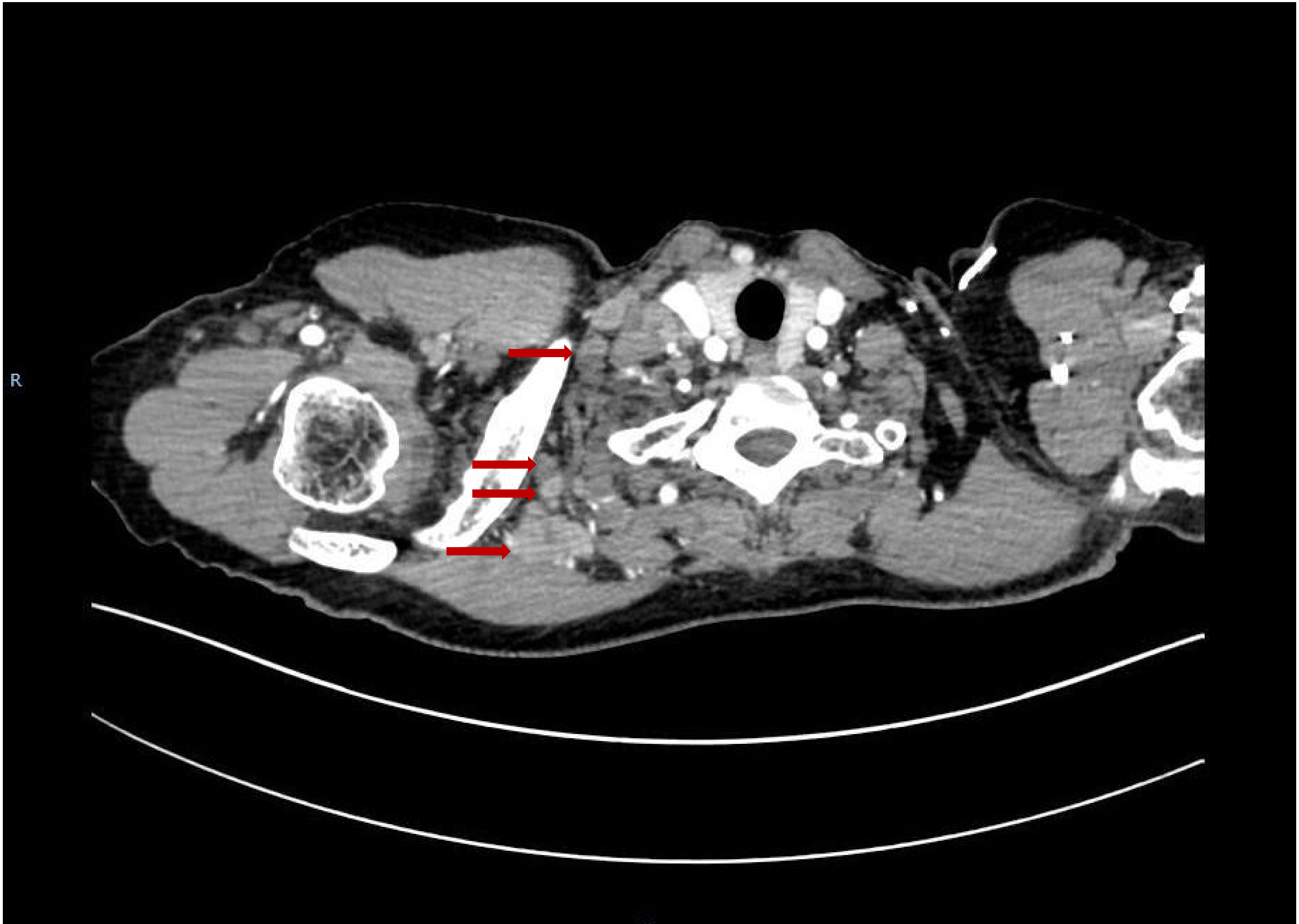
R

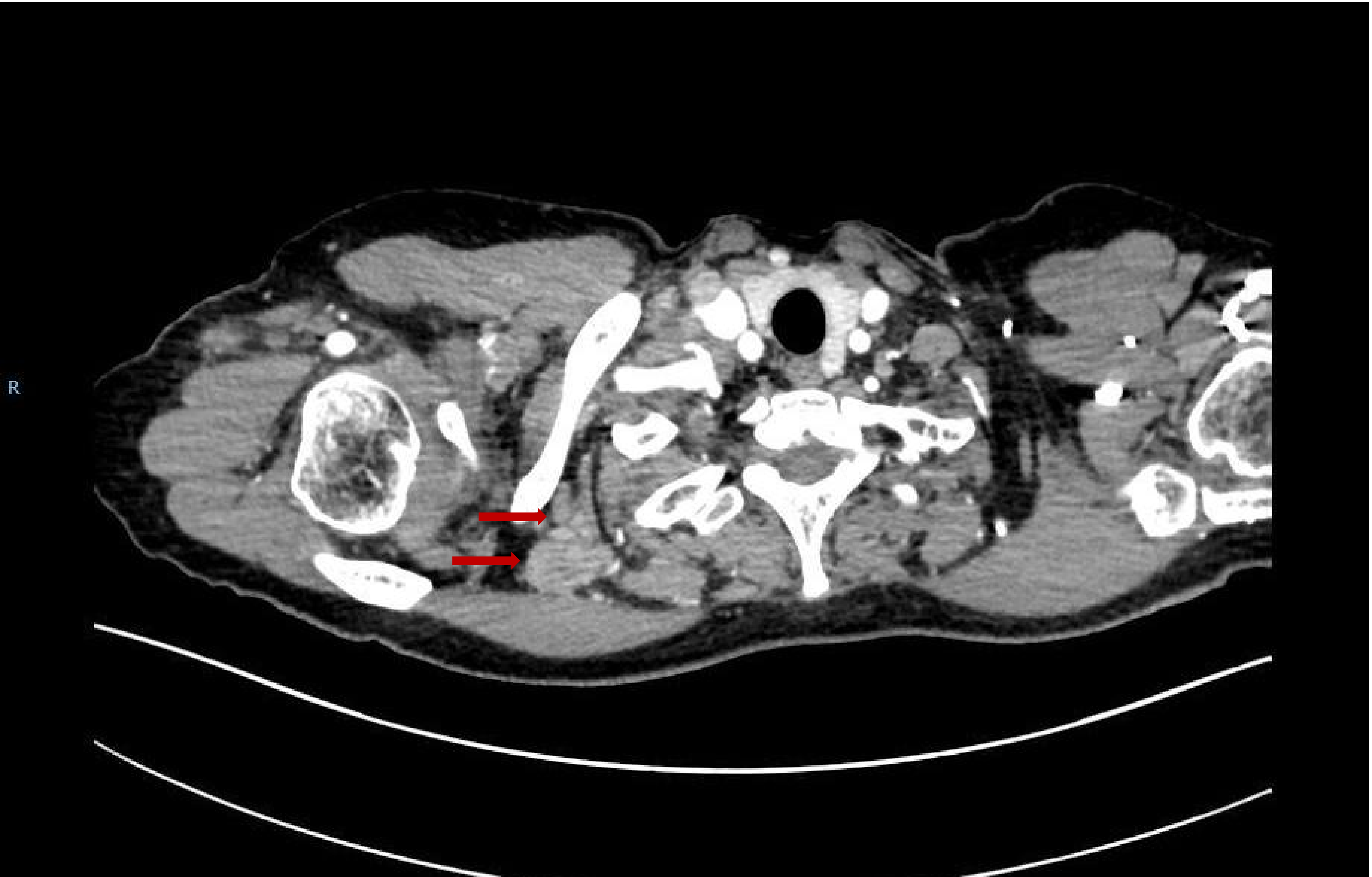
R

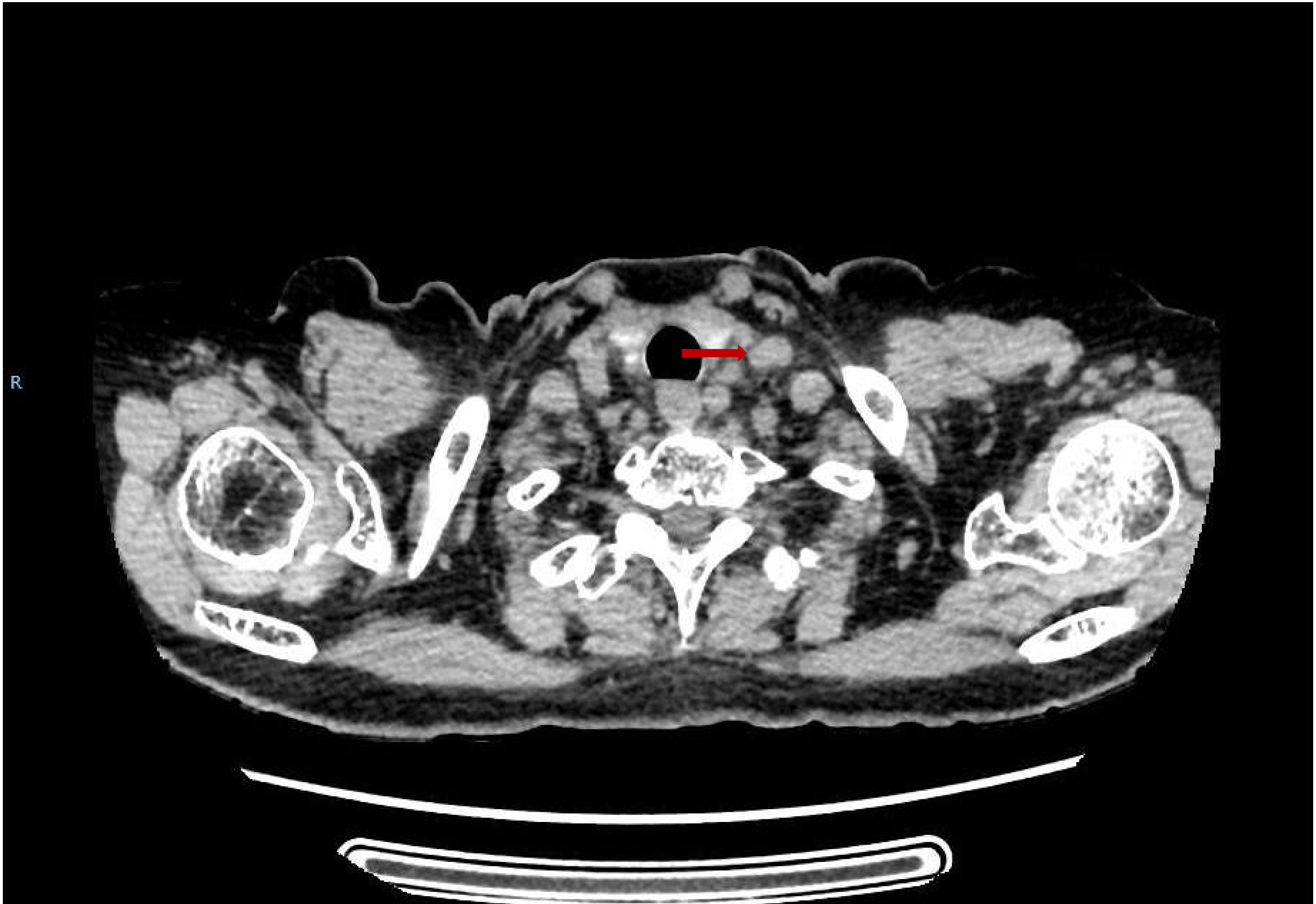
R

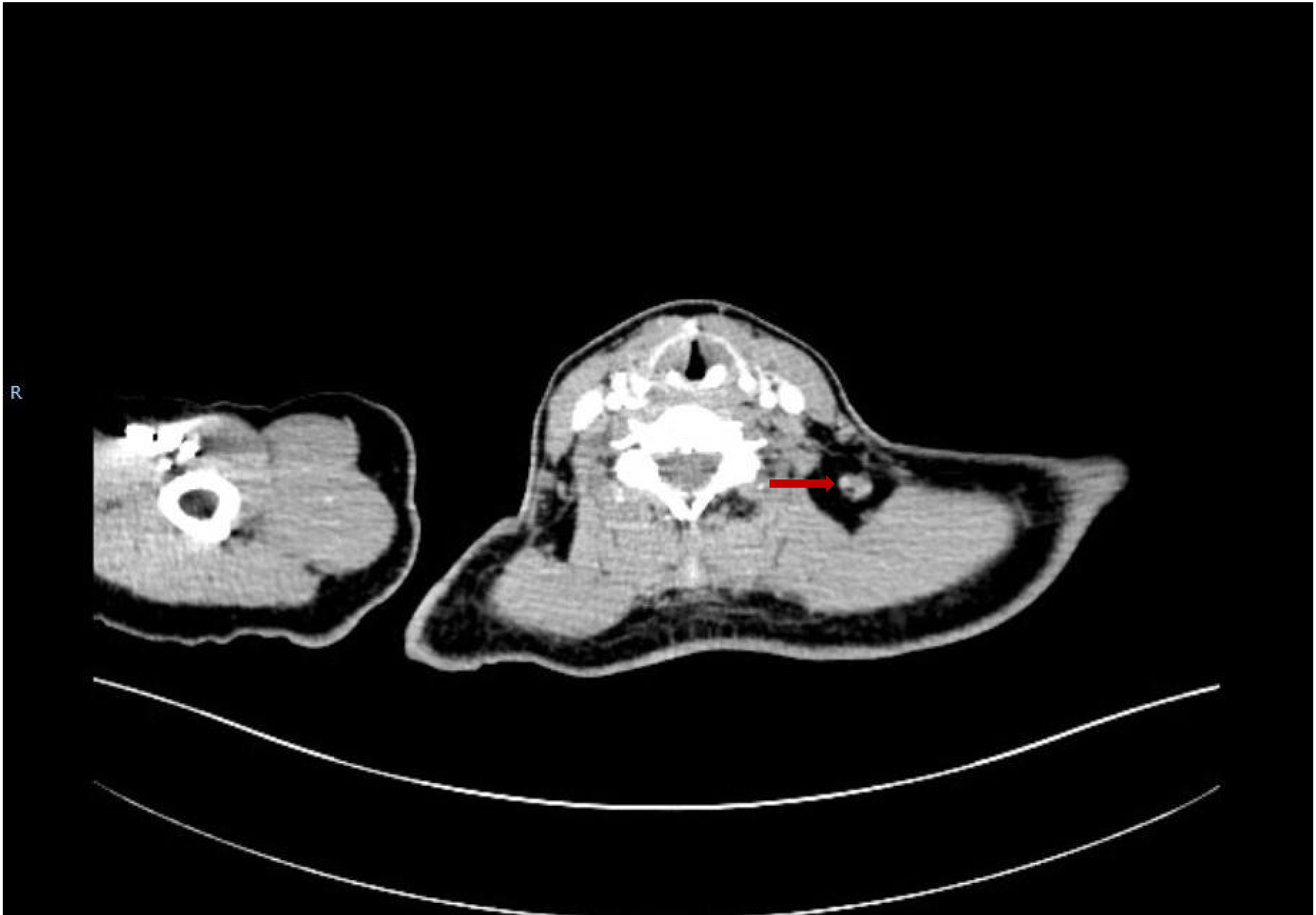
R

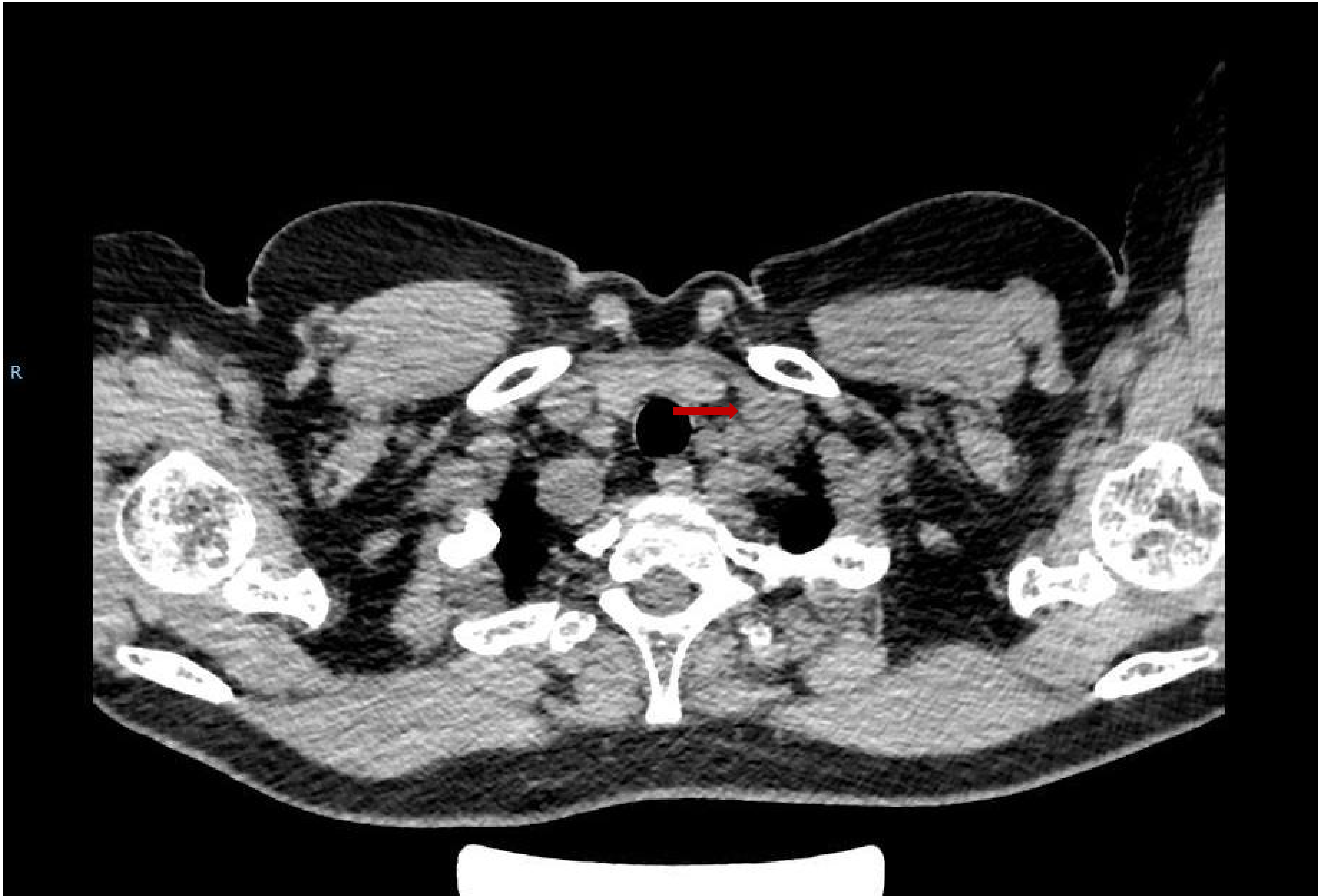
R

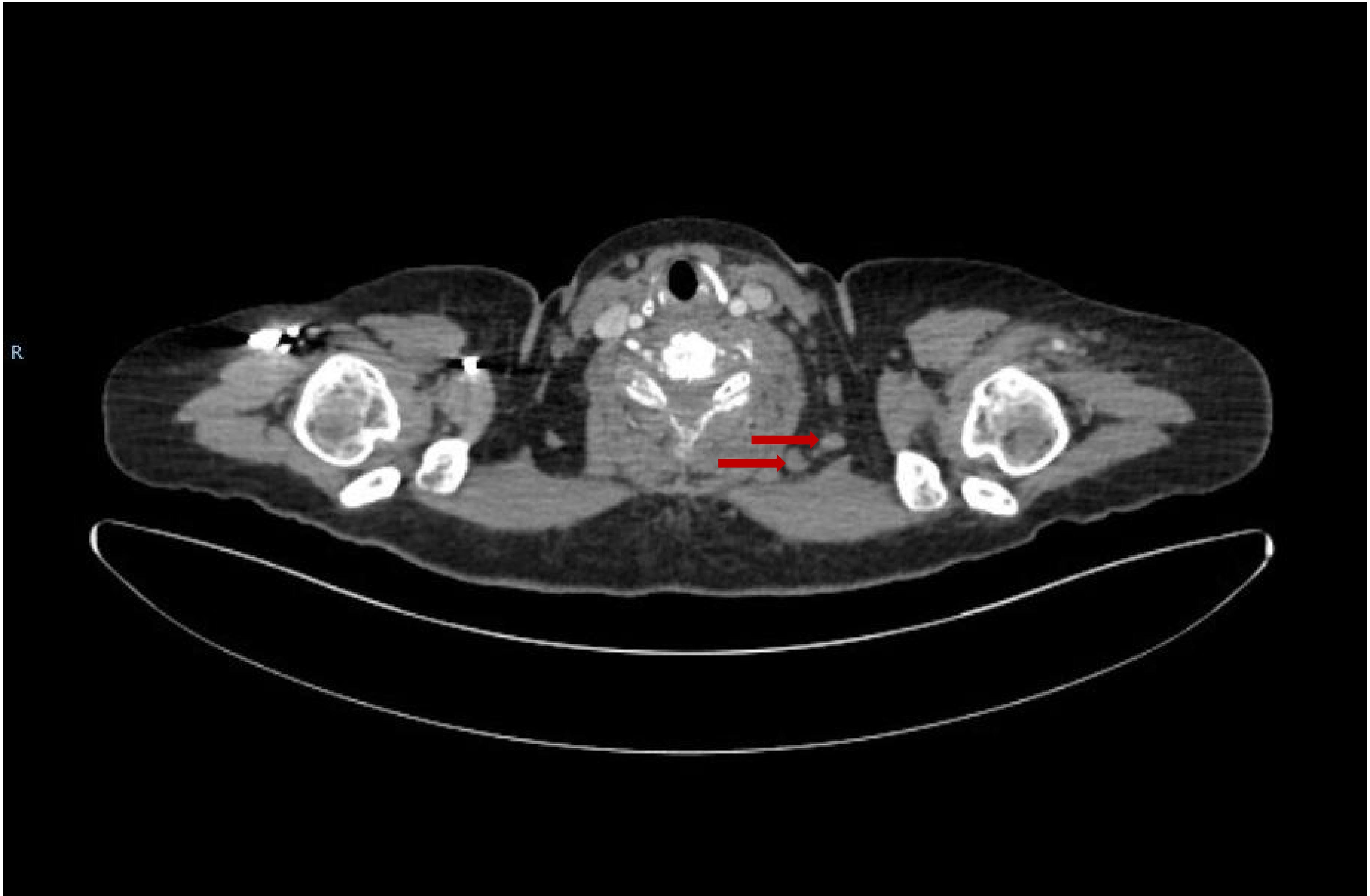
R

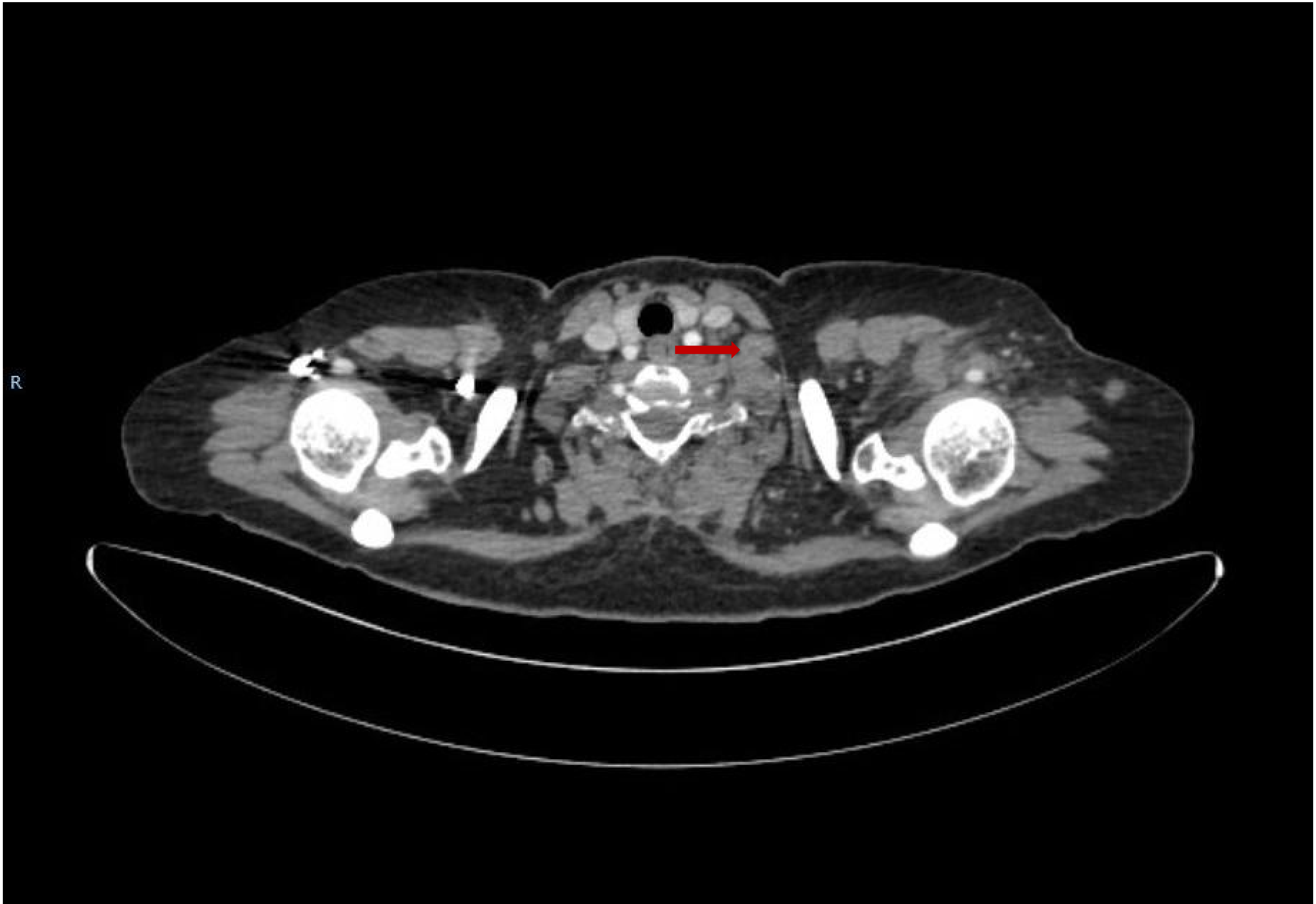
R

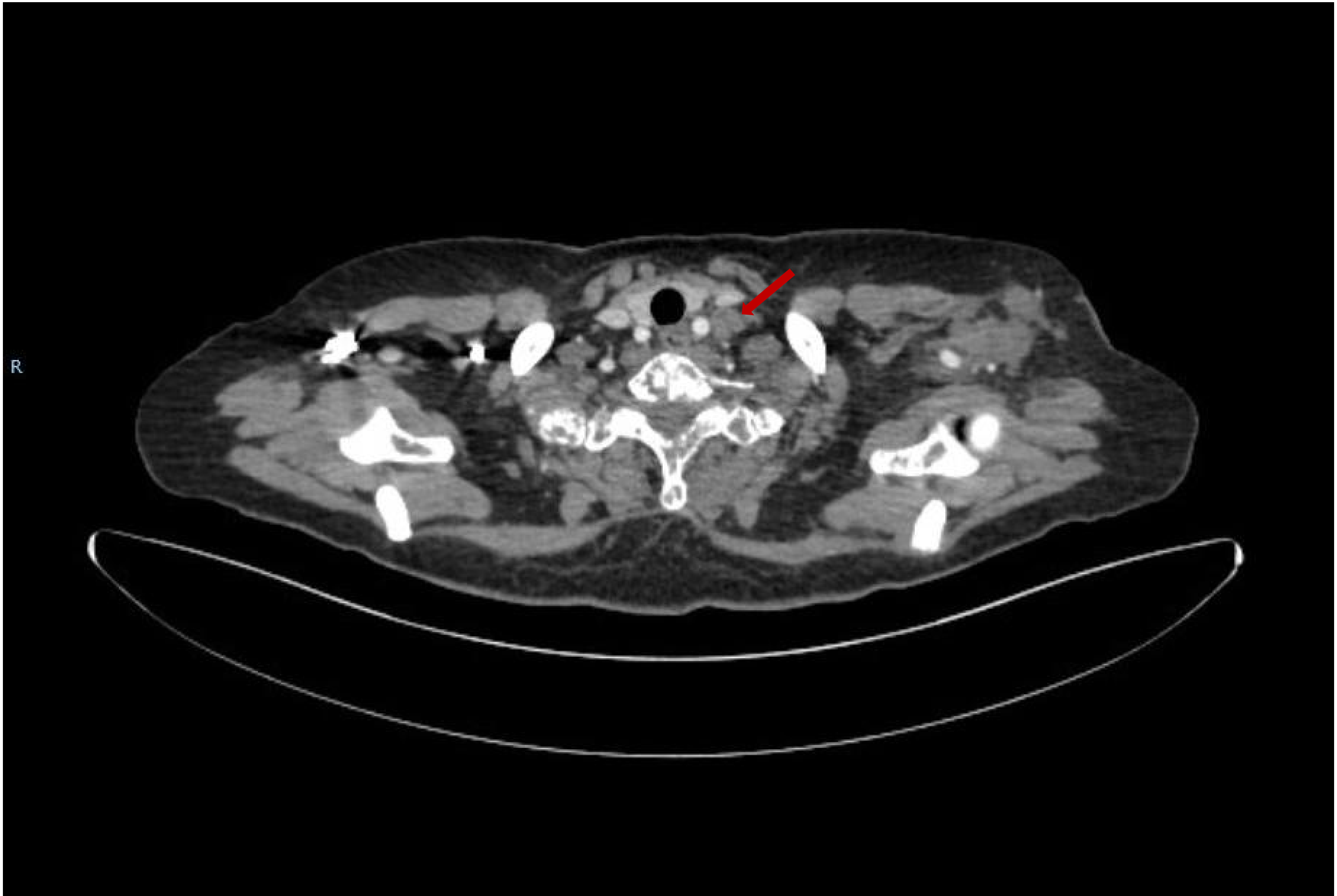
R

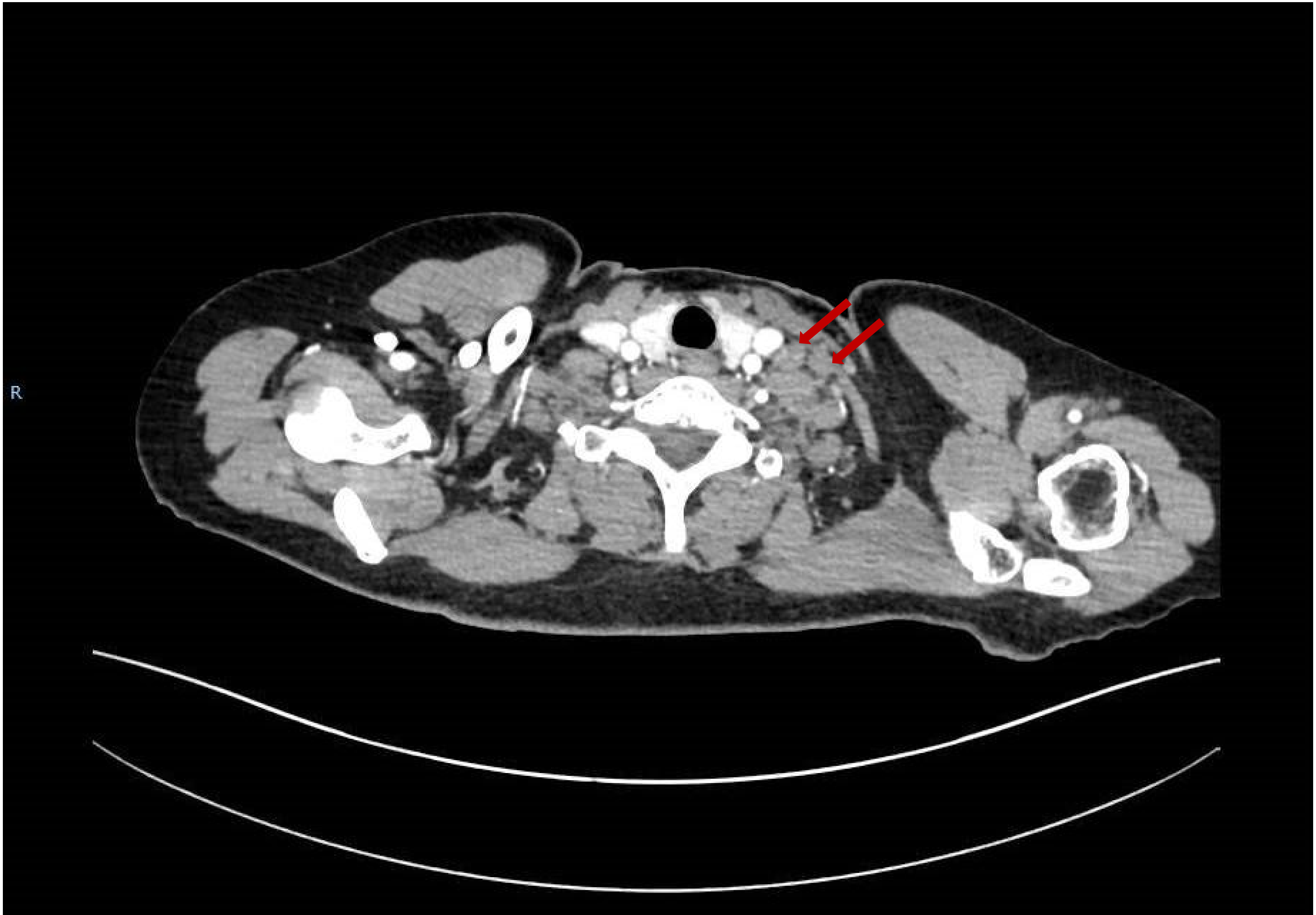
R

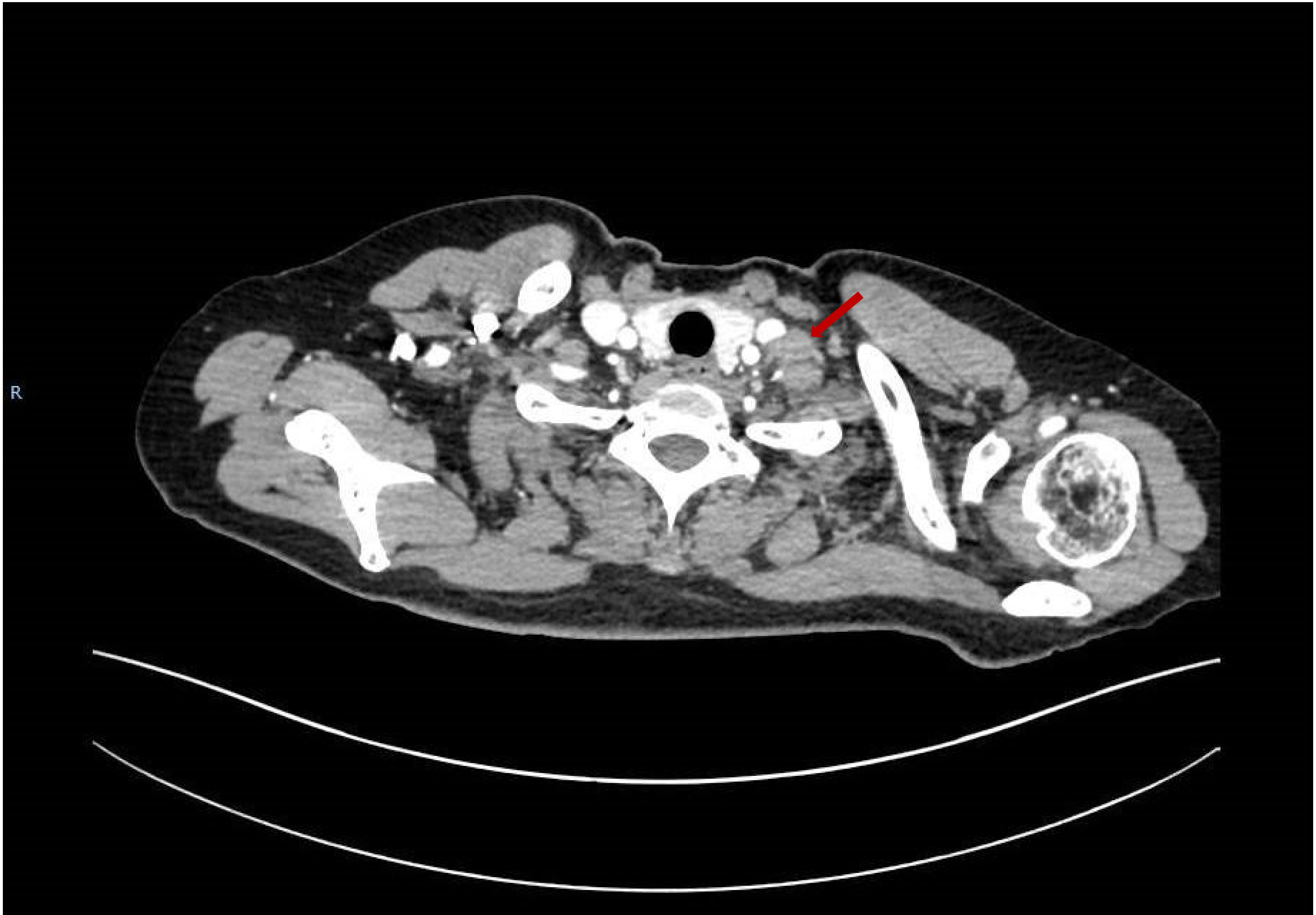
R

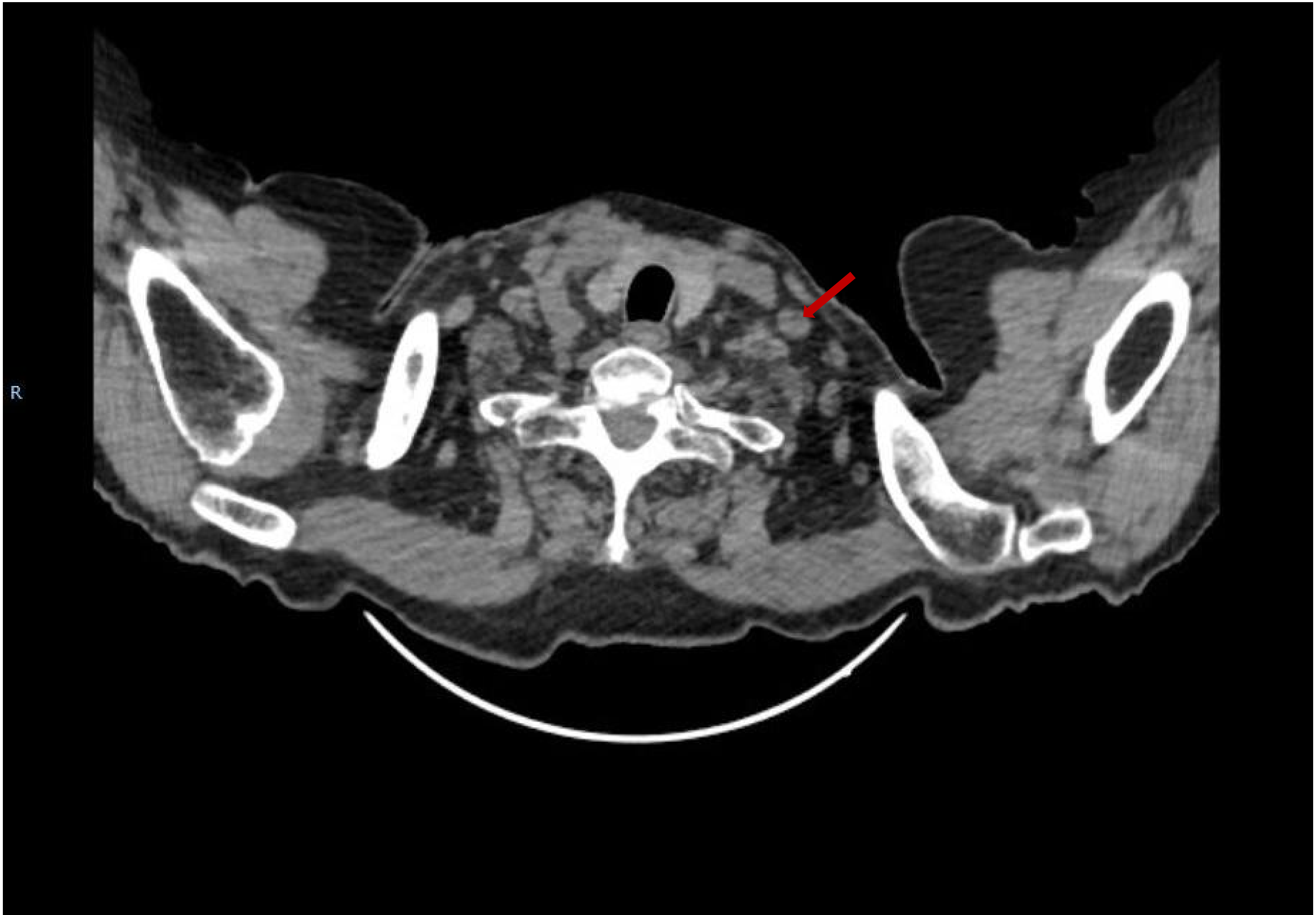

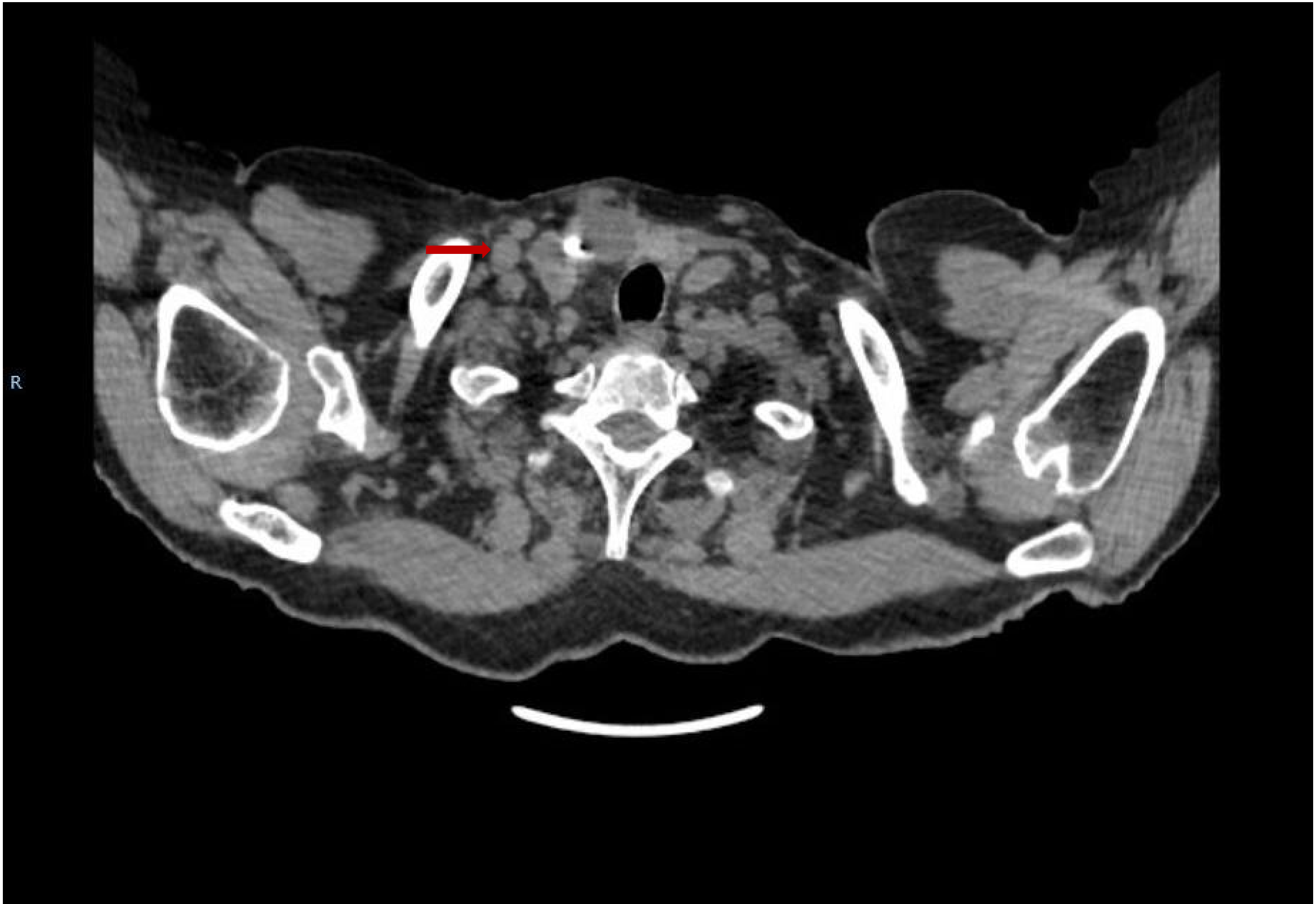
R

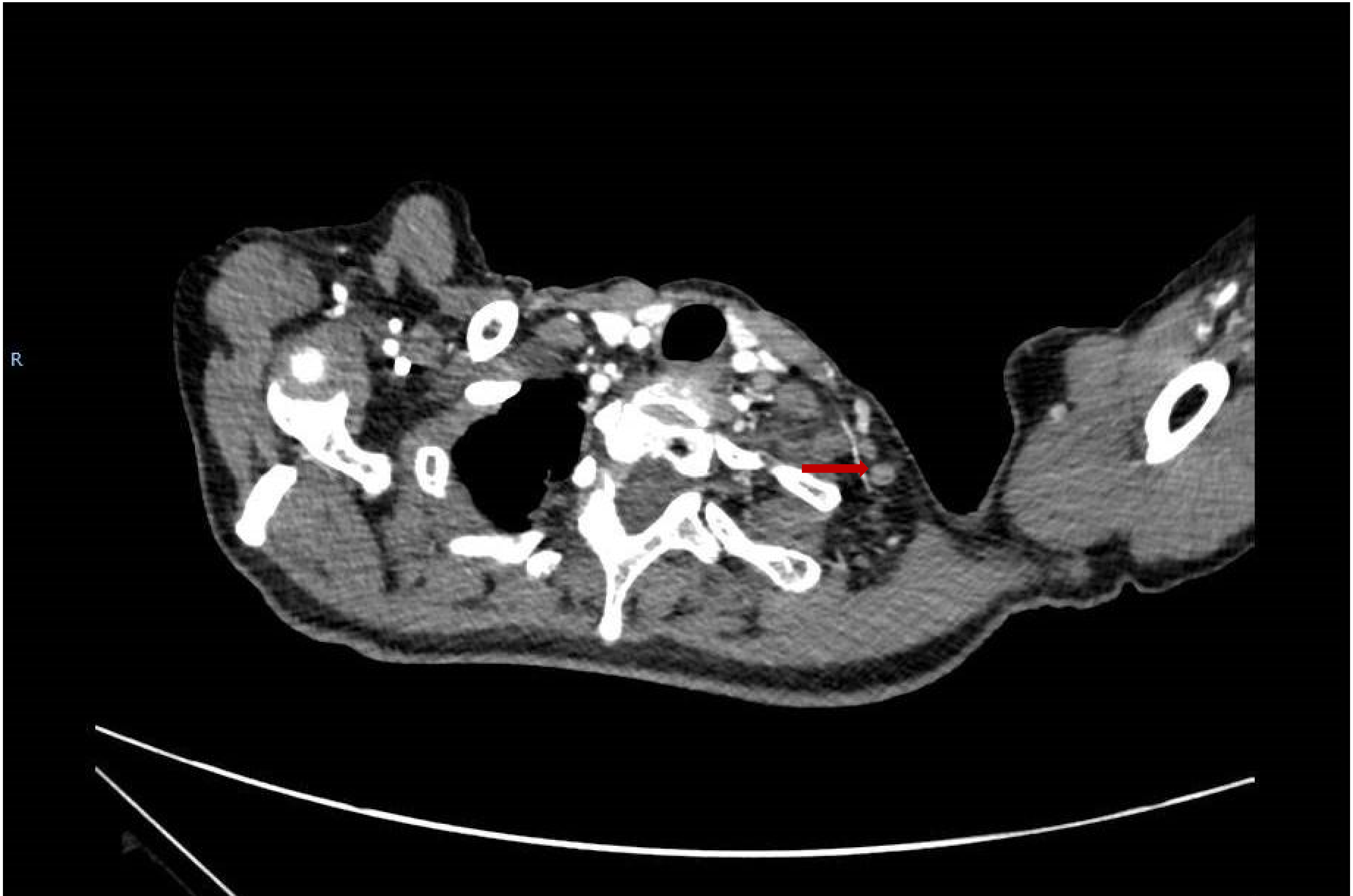
R

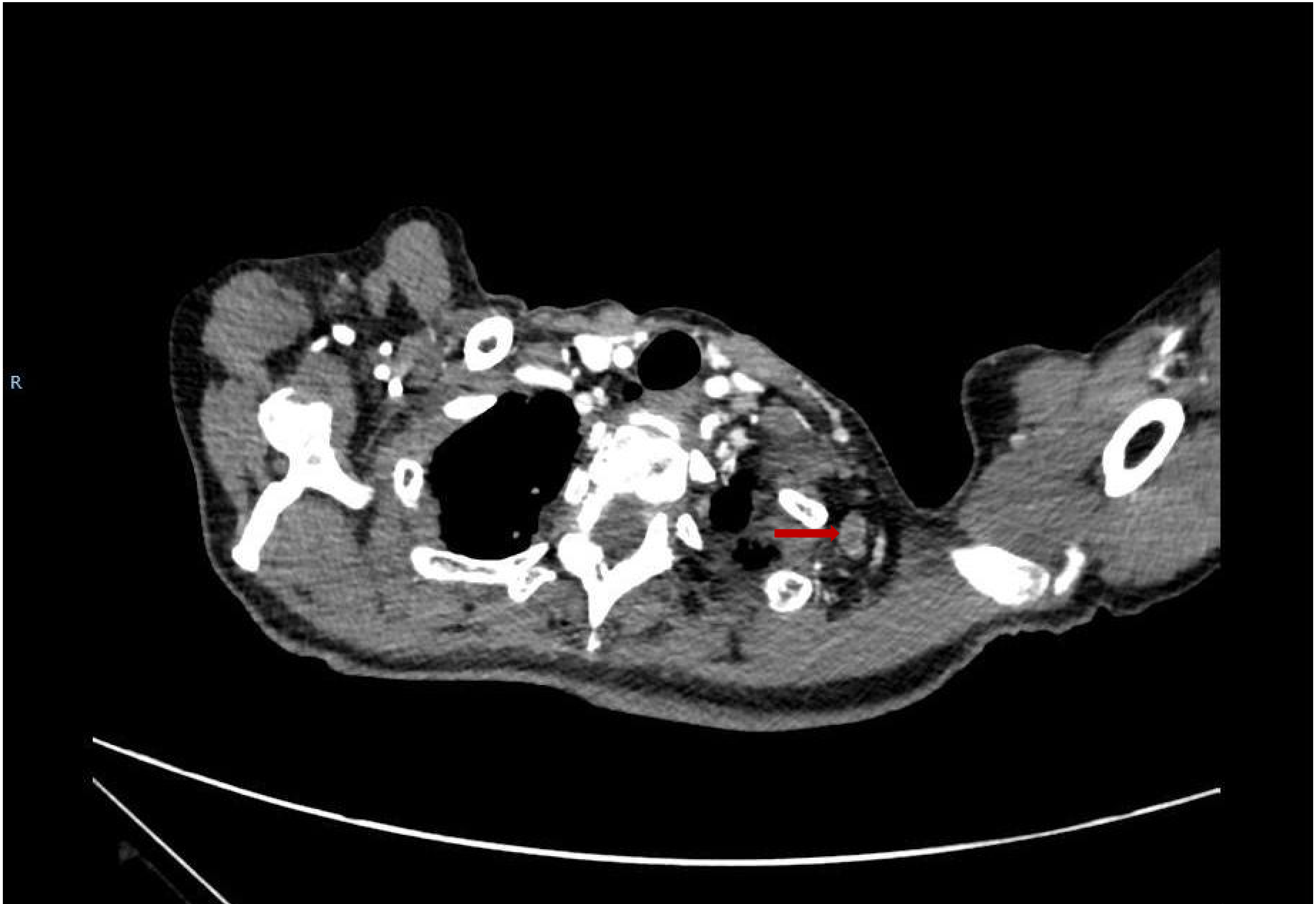
R

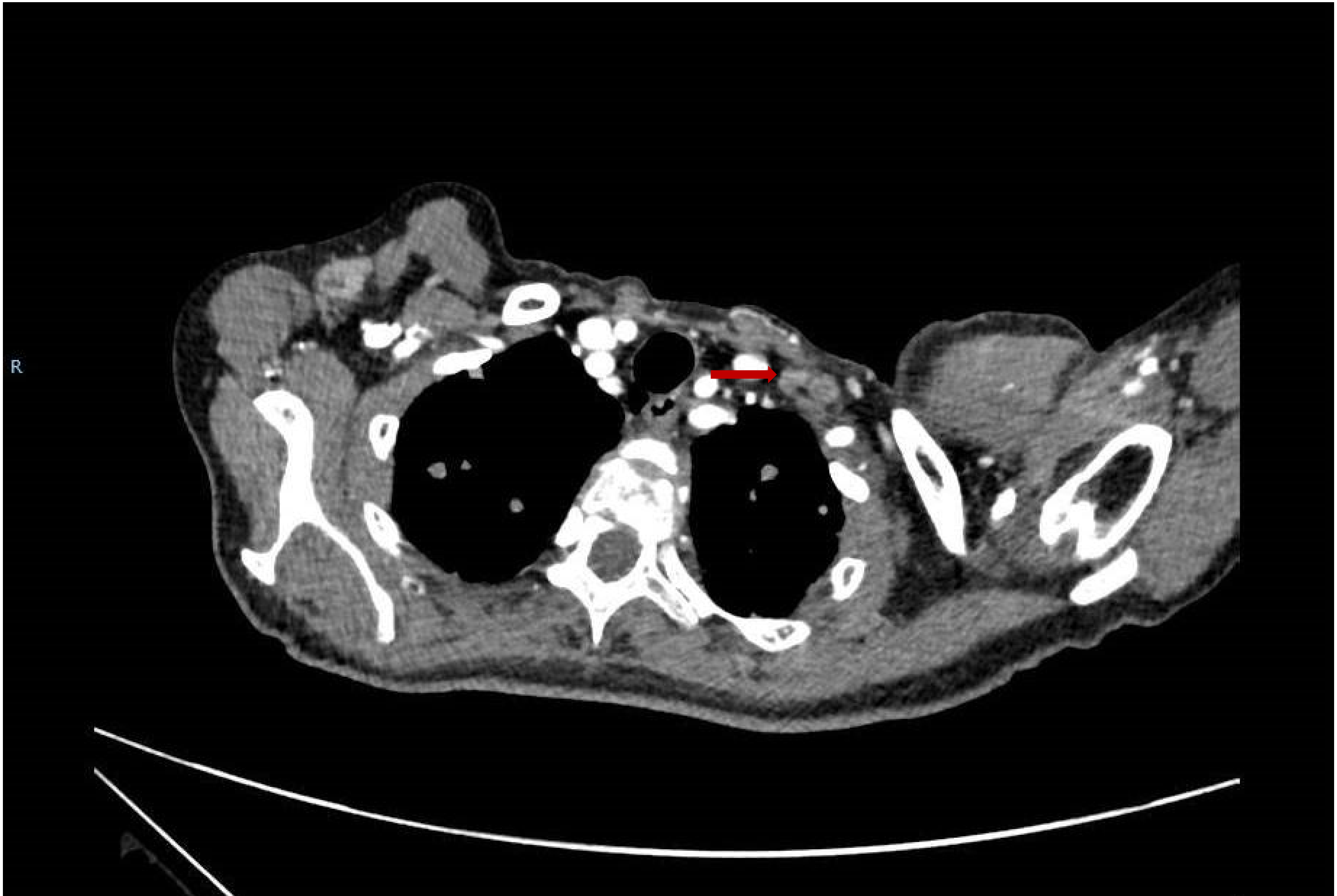
R

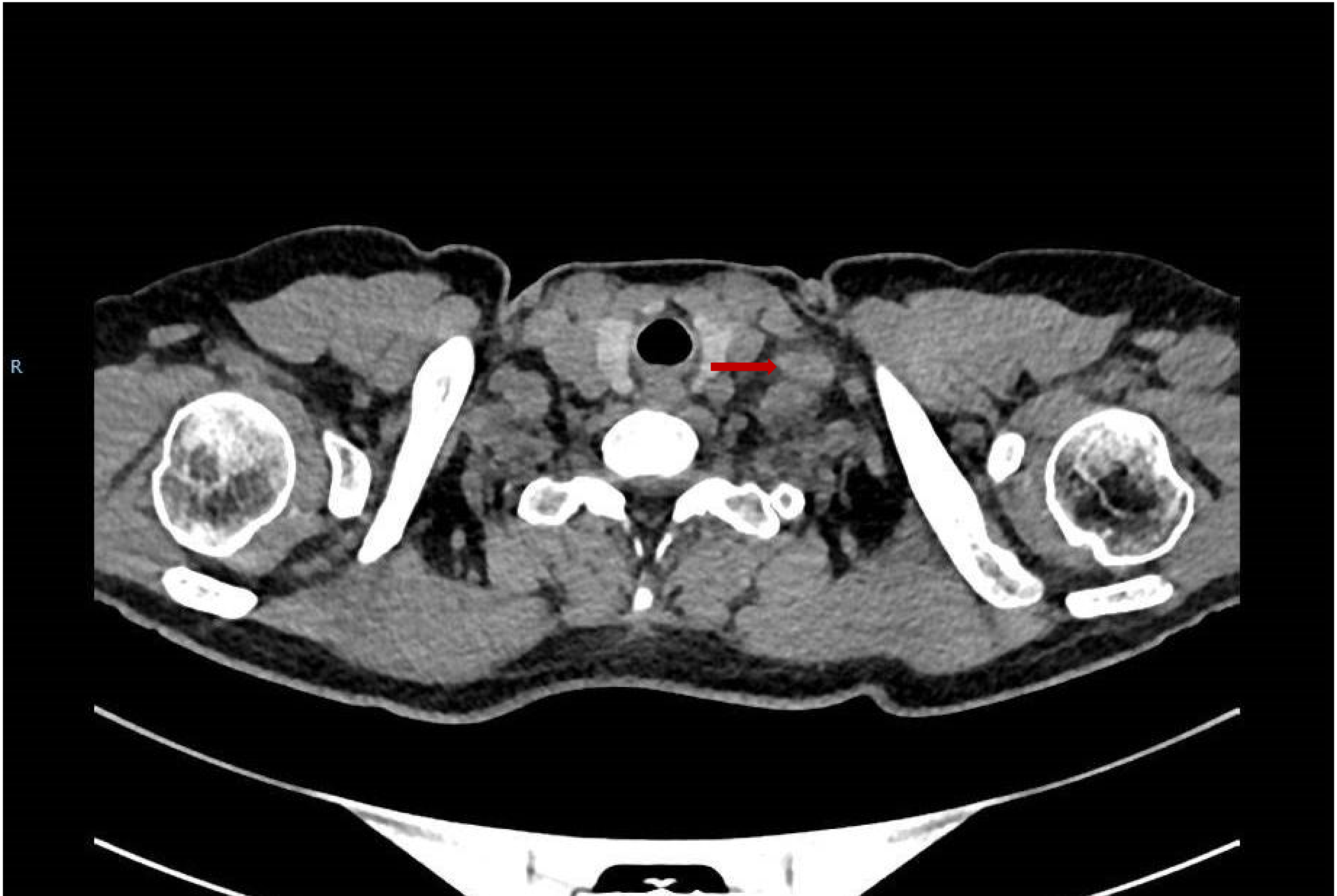
R
